# Metabolomic Quantitative Trait Loci (mQTL) Mapping Implicates the Ubiquitin Proteasome System in Cardiovascular Disease Pathogenesis

**DOI:** 10.1371/journal.pgen.1005553

**Published:** 2015-11-05

**Authors:** William E. Kraus, Deborah M. Muoio, Robert Stevens, Damian Craig, James R. Bain, Elizabeth Grass, Carol Haynes, Lydia Kwee, Xuejun Qin, Dorothy H. Slentz, Deidre Krupp, Michael Muehlbauer, Elizabeth R. Hauser, Simon G. Gregory, Christopher B. Newgard, Svati H. Shah

**Affiliations:** 1 Division of Cardiology, Department of Medicine, Duke University, Durham, North Carolina, United States of America; 2 Duke Molecular Physiology Institute, Duke University, Durham, North Carolina, United States of America; 3 Division of Endocrinology, Department of Medicine, Duke University, Durham, North Carolina, United States of America; 4 Department of Biostatistics and Bioinformatics, Duke University, Durham, North Carolina, United States of America; University of California, Los Angeles School of Medicine, UNITED STATES

## Abstract

Levels of certain circulating short-chain dicarboxylacylcarnitine (SCDA), long-chain dicarboxylacylcarnitine (LCDA) and medium chain acylcarnitine (MCA) metabolites are heritable and predict cardiovascular disease (CVD) events. Little is known about the biological pathways that influence levels of most of these metabolites. Here, we analyzed genetics, epigenetics, and transcriptomics with metabolomics in samples from a large CVD cohort to identify novel genetic markers for CVD and to better understand the role of metabolites in CVD pathogenesis. Using genomewide association in the CATHGEN cohort (*N* = 1490), we observed associations of several metabolites with genetic loci. Our strongest findings were for SCDA metabolite levels with variants in genes that regulate components of endoplasmic reticulum (ER) stress (*USP3*, *HERC1*, *STIM1*, *SEL1L*, *FBXO25*, *SUGT1*) These findings were validated in a second cohort of CATHGEN subjects (*N* = 2022, combined p = 8.4x10^-6^–2.3x10^-10^). Importantly, variants in these genes independently predicted CVD events. Association of genomewide methylation profiles with SCDA metabolites identified two ER stress genes as differentially methylated (*BRSK2* and *HOOK2*). Expression quantitative trait loci (eQTL) pathway analyses driven by gene variants and SCDA metabolites corroborated perturbations in ER stress and highlighted the ubiquitin proteasome system (UPS) arm. Moreover, culture of human kidney cells in the presence of levels of fatty acids found in individuals with cardiometabolic disease, induced accumulation of SCDA metabolites in parallel with increases in the ER stress marker BiP. Thus, our integrative strategy implicates the UPS arm of the ER stress pathway in CVD pathogenesis, and identifies novel genetic loci associated with CVD event risk.

## Author Summary

Cardiovascular disease is a strongly heritable trait. Despite application of the latest genomic technologies, the genetic architecture of disease risk remains poorly defined, and mechanisms underlying this susceptibility are incompletely understood. In this study, we performed genome-wide mapping of heart disease-related metabolites measured in the blood as the genetic traits of interest (instead of the disease itself), in a large cohort of 3512 patients at risk of heart disease from the CATHGEN study. Our goal was to discover new cardiovascular disease genes and thereby mechanisms of disease pathogenesis by understanding the genes that regulate levels of these metabolites. These analyses identified novel genetic variants associated with metabolite levels and with cardiovascular disease itself. Importantly, by utilizing an unbiased systems-based approach integrating genetics, gene expression, epigenetics and metabolomics, we uncovered a novel pathway of heart disease pathogenesis, that of endoplasmic reticulum (ER) stress, represented by elevated levels of circulating short-chain dicarboxylacylcarnitine (SCDA) metabolites.

## Introduction

Despite the strong heritability of cardiovascular disease (CVD), its underlying genetic architecture remains incompletely characterized. Genomewide association studies (GWAS) have converged on association of CVD with a locus on chromosome 9p21 [1], but the variants confer modest risk and are of unclear functional significance. One limitation of GWAS studies for complex diseases is the search for association with disease as a binary endpoint, rather than with molecular markers that define risk. An alternative approach is to search for variations in the genome that associate with variation in complex traits. In fact, many diseases can be defined by an underlying quantitative scale, and these “intermediate” traits may have a stronger functional relationship to the causative gene, thereby providing a stronger signal for the disease process. Metabolite levels measured by the emerging tools of metabolomics may be particularly useful for such studies. Indeed, integration of GWAS with metabolomic profiles in population-based cohorts [2] has demonstrated that as much as 12% of variance in metabolite levels is determined by single nucleotide polymorphisms (SNPs). However, most studies of this type performed to date have not used disease-burdened cohorts, so clear linkages between genetic signals, intermediate phenotypes and disease remain to be discovered.

Metabolomic profiling has identified novel biomarkers for CVD risk [3–5]. For example, a cluster of heritable [6] short-chain dicarboxylacylcarnitine (SCDA) metabolites measured in plasma (comprised of the mono-carnitine esters of short-chain, *alpha-*, *omega-*diacids), a cluster of long-chain dicarboxylacylcarnitines (LCDA), and a cluster of medium-chain acylcarnitines (MCA) predict CVD events in cardiovascular cohorts [4, 5], in patients undergoing coronary artery bypass grafting [3], and add incremental risk prediction to robust clinical models inclusive of >20 variables [5]. Little is known about the biological pathways represented by these metabolites and how they may predispose to CVD. Thus, we hypothesized that integration of metabolomics with genetics, epigenetics, and transcriptomics could define novel mechanisms of CVD pathogenesis by identifying metabolic quantitative trait loci (mQTL) that are CVD risk factors. We performed a GWAS of metabolite levels in a large cardiovascular cohort referred for cardiac catheterization (CATHGEN, *N* = 1490) and validated our findings in a second cohort (CATHGEN, *N* = 2022). A proportion of study subjects (44%) did not have clinically significant atherosclerotic coronary artery disease at time of catheterization; regardless, all individuals were analyzed given that metabolites predict risk of CVD events even in individuals without coronary artery disease, and because these individuals are still at risk for these events. We found that genetic loci that strongly associate with SCDA levels also predict incident CVD events, and are linked to ER stress. Genes differentially methylated in subjects at the extremes of SCDA levels also report on ER stress. Gene expression quantitative trait loci (eQTL) pathway analysis identified ER stress as an expression module associated with disease risk, particularly highlighting the ubiquitin proteasome system (UPS) arm of ER stress. Thus, this multi-platform “omics” approach identified a molecular pathway (ER stress and dysregulation of the UPS) associated with a prevalent complex disease.

## Results

Table 1 displays baseline characteristics of the study population. PCA of metabolomic data identified 14 factors with metabolites in each factor clustering within biochemical pathways (S1 Table), and clustering similar to our previous studies [3–5, 7]. For this study, we performed GWAS using the top three PCA-derived factors: factor 1 (composed of MCA metabolites), factor 2 (composed of LCDA metabolites), and factor 3 (composed of SCDA metabolites), all of which we have previously identified as predicting CVD events (S2 Table) [3–5]. S1 Fig details the overall study flow.

**Table 1:** Baseline characteristics of study population.

	Discovery (N = 1490)	Validation (N = 2022)
Age, mean (SD)	57.6 (11.6)	62.2 (11.9)
Race		
% White	68%	72%
% Black	21%	21%
Sex (% female)	48.7%	38.2%
BMI, mean (SD)	30.8 (7.9)	30.1 (7.0)
Ejection fraction, mean (SD)	58.7 (11.4)	56.5 (13.5)
Creatinine, mean (SD)	1.1 (0.9)	1.3 (1.5)
Diabetes (%)	28.0%	31.2%
Hyperlipidemia (%)	56.7%	59.4%
Hypertension (%)	67.4%	67.4%
Smoking (%)	51.2%	47.2%
Family history (%)	38.3%	36.2%
Number of diseased coronary arteries		
0	50.9%	39.0%
1	16.7%	23.6%
2	15.1%	16.0%
3	17.3%	21.4%
Heart failure (%)	20.1%	26.6%
Renal disease (%)	1.2%	2.5%

### Metabolic quantitative trait loci (mQTL) of metabolite levels reside in genes reporting on ER stress

**Fig 1:**
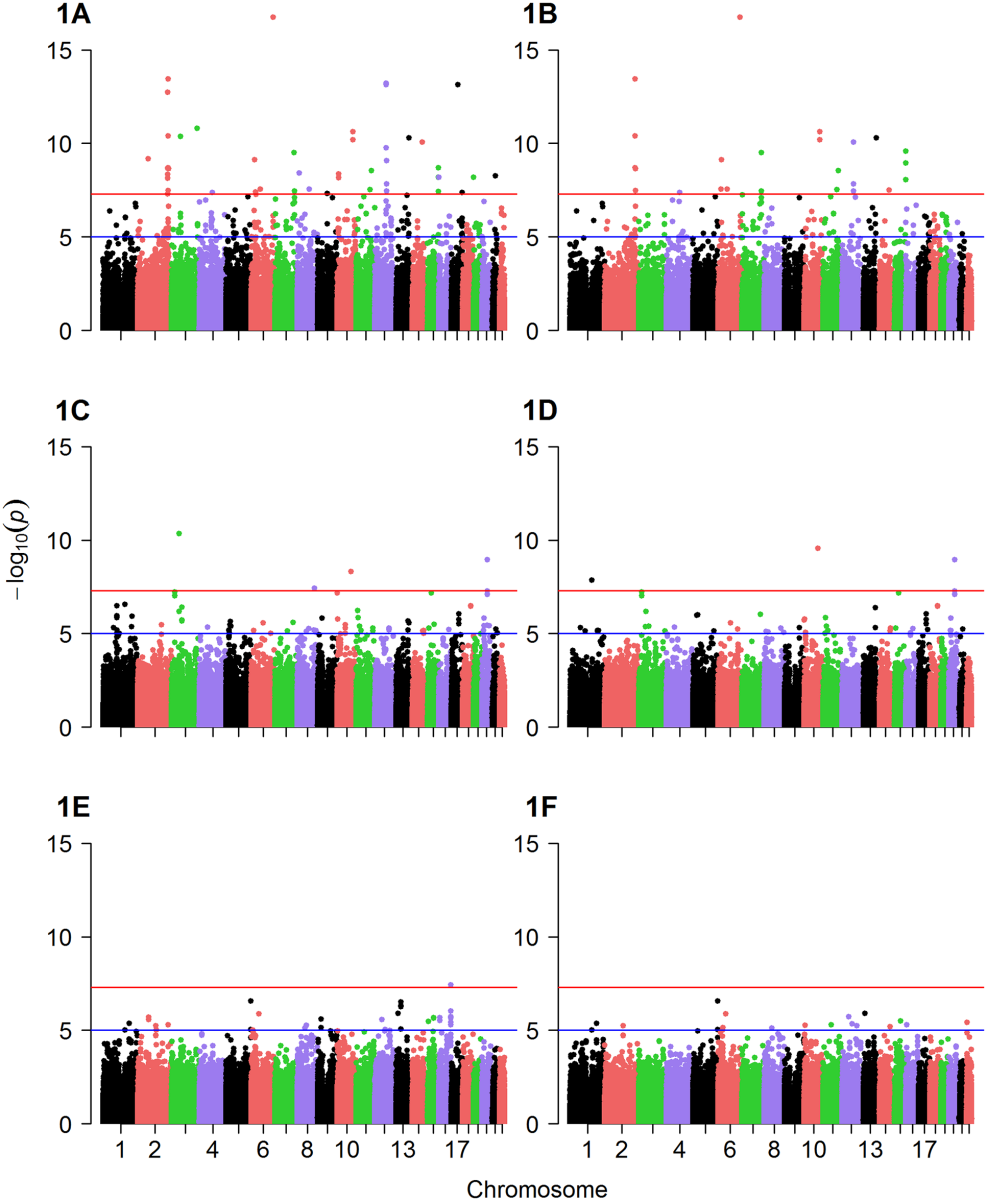
Manhattan plots of GWAS results. Displayed are Manhattan plots of the association results for GWAS (discovery cohort, whites only) with (A) factor 1 additive model, (B) factor 1 dominant model, (C) factor 2 additive model, (D) factor 2 dominant model, (E) factor 3 additive model and (F) factor 3 dominant model.

Factor 1, factor 2 and factor 3 scores were used as the quantitative traits in GWAS analysis to identify mQTL. Q-Q plots suggested the presence of SNPs associated with levels of each of the three metabolite factors (S2, S3 and S4 Figs). Several SNPs were significantly associated with metabolite factor levels at genomewide significance (p≤10^−6^) in additive models in the discovery cohort (Fig 1A–1F) and confirmed (p≤0.05) in the validation cohort (Table 2). Specifically, eight SNPs were associated with factor 1 (MCA) levels in any race, but with only two of these SNPs showing more than nominal significance in the validation cohort (Table 2): rs10987728 (in cyclin dependent kinase 9 [*CDK9*]) and rs6738286 (intergenic between transition protein 1 [*TNP1*] and disrupted in renal carcinoma 3 [*DIRC3*]). Twelve SNPs were associated with factor 2 (LCDA) levels in any race (Table 2), with only two of them showing more than nominal significance in the validation cohort (rs12129555 just downstream from polymeric immunoglobulin receptor [*PIGR*] and rs17025690 in Usher syndrome 2A [*USH2a*]). Factor 3 (SCDA) showed the strongest mQTL with twelve SNPs being associated with SCDA levels in any race (Table 2), and four of these SNPs showing more than nominal significance in the validation cohort: rs2228513 in HERC1 HECT and RLD domain containing E3 ubiquitin protein ligase family member 1 (*HERC1*), rs10450989 in ubiquitin specific protease 3 (*USP3*), rs11771619 in round spermatid basic protein 1-like (*RSBN1L*), and rs1869075 (intergenic between F-box protein 25 [*FBXO25*] and glutamate rich 1 [*ERICH1*]). Effect sizes (β, i.e. per 1 unit change in factor levels) ranged from to -0.38 to 2.17 (factor 1), -0.19 to 1.16 (factor 2), and -0.43 to 1.72 (factor 3).

**Table 2:** Significant mQTL from GWAS of metabolite factors 1, 2 and 3. Presented are SNPs meeting genomewide significance for association with factor 1 (MCA metabolites), factor 2 (LCDA metabolites) and factor 3 (SCDA metabolites) in race-stratified analyses in the discovery cohort (p≤10^−6^) also showing nominal association (p≤0.05) in the validation cohort, ranked by meta-analysis p-value.

Gene	Factor	SNP	Chr:Pos	MAF	Model	Race^a^	Discovery p^b^	Validation p^b^	Meta p^c^
*HERC1*	3	rs2228513	15:63950887	0.05	Add	W	2.2x10^-6^	3.2x10^-6^	7.9x10^-10^
*HERC1*	3	rs2228513	15:63950887	0.05	Dom	W	3.2x10^-6^	1.2x10^-3^	5.0x10^-8^
*USP3*	3	rs10450989	15:63846508	0.05	Add	W	2.2x10^-6^	3.1x10^-6^	2.3x10^-10^
*USP3*	3	rs10450989	15:63846508	0.05	Dom	W	3.1x10^-6^	4.7x10^-4^	1.6x10^-8^
*PIGR*	2	rs12129555	1:207101264	0.03	Add	B	2.4x10^-7^	2.5x10^-3^	2.1x10^-8^
*PIGR*	2	rs12129555	1:207101264	0.03	Dom	B	2.4x10^-7^	1.2x10^-3^	7.6x10^-9^
*LOC100289596|ZNF521*	2	rs4800615	18:22622445	0.03	Add	B	1.6x10^-9^	0.03	3.0x10^-8^
*LOC100289596|ZNF521*	2	rs4800615	18:22622445	0.03	Dom	B	1.6x10^-9^	0.04	6.8x10^-8^
*ZNF521*	2	rs12965721	18:22648924	0.05	Add	B	2x10^-8^	0.02	8.8x10^-8^
*CDK9*	1	rs10987728	9:130553040	0.01	Add	W	5.6x10^-6^	1.2x10^-3^	7.4x10^-8^
*CDK9*	1	rs10987728	9:130553040	0.01	Dom	W	5.6x10^-6^	9.3x10^-4^	5.7x10^-8^
*USH2A*	2	rs17025690	1:216119893	0.04	Add	B	7.9x10^-7^	5.4x10^-3^	1.4x10^-7^
*RSBN1L*	3	rs11771619	7:77403278	0.02	Add	B	2.3x10^-6^	7.6x10^-3^	4.4x10^-7^
*RPL36AP40*	2	rs9633819	11:25529987	0.03	Add	B	7x10^-7^	0.04	2.3x10^-6^
*RPL36AP40*	2	rs9633819	11:25529987	0.03	Dom	B	7x10^-7^	0.02	8.6x10^-7^
*NFIA|TM2D1*	2	rs17122575	1:62104766	0.06	Add	B	2.9x10^-7^	0.01	2.6x10^-7^
*TNP1|DIRC3*	1	rs6738286	2:217994269	0.02	Add	W	4.5x10^-6^	3.5x10^-3^	2.6x10^-7^
*TNP1|DIRC3*	1	rs6738286	2:217994269	0.02	Dom	W	4.5x10^-6^	3.5x10^-3^	2.6x10^-7^
*FBXO25*|*ERICH1*	3	rs1869075	8:540949	0.10	Add	B	3.2x10^-6^	7.1x10^-3^	5x10^-7^
*PTPRT*	2	rs6016673	20:40693779	0.02	Dom	W	8.3x10^-8^	0.04	7.5x10^-7^
*OLFM4|SUGT1*	3	rs17573278	13:53995627	0.05	Add	W	4.8x10^-7^	0.03	1.2x10^-6^
*OLFM4|SUGT1*	3	rs9591507	13:53929144	0.05	Add	W	5.4x10^-7^	0.03	1.1x10^-6^
*FER1L6|TMEM65*	2	rs7816704	8:125263468	0.08	Add	B	6.1x10^-7^	0.03	1.2x10^-6^
*COL23A1*	3	rs17081346	5:177895383	0.01	Add	W	2.8x10^-7^	0.04	1.3x10^-6^
*COL23A1*	3	rs17081346	5:177895383	0.01	Dom	W	2.8x10^-7^	0.04	1.3x10^-6^
*COL23A1*	3	rs17052428	5:177898958	0.01	Add	W	2.8x10^-7^	0.04	1.3x10^-6^
*COL23A1*	3	rs17052428	5:177898958	0.01	Dom	W	2.8x10^-7^	0.04	1.3x10^-6^
*OLFM4|SUGT1*	3	rs9285184	13:53977134	0.05	Add	W	3.1x10^-7^	0.04	1.6x10^-6^
*CACNA2D2*	1	rs41291734	3:50513613	0.03	Add	W	5.8x10^-6^	0.03	4.7x10^-6^
*CACNA2D2*	1	rs41291734	3:50513613	0.03	Dom	W	2.5x10^-6^	0.02	2.2x10^-6^
*PDGFD*	2	rs12421553	11:103838440	0.21	Add	B	7.5x10^-7^	0.02	1x10^-6^
*EBF2|PPP2R2A*	1	rs2170483	8:26133566	0.04	Add	W	2.4x10^-6^	0.03	3.2x10^-6^
*EBF2|PPP2R2A*	1	rs2170483	8:26133566	0.04	Dom	W	2.4x10^-6^	0.03	3.7x10^-6^
*ELF3|GPR37L1*	3	rs12139192	1:202003269	0.06	Add	B	2.7x10^-6^	0.04	6.1x10^-6^
*ELF3|GPR37L1*	3	rs12139192	1:202003269	0.06	Dom	B	2.7x10^-6^	0.02	1.8x10^-6^
*SFTA1P*	2	rs17148556	10:10676274	0.03	Add	B	2.3x10^-7^	0.04	1.1x10^-6^
*SFTA1P*	2	rs17148556	10:10676274	0.03	Dom	B	2.3x10^-7^	0.04	1.3x10^-6^
*ZNF267*	2	rs4889565	16:31897308	0.04	Dom	W	9.1x10^-6^	0.02	4.3x10^-6^
*MACROD2*	2	rs2423983	20:15709520	0.08	Dom	W	6.8x10^-6^	0.03	5.1x10^-6^
*PLA2G4A|FAM5C*	3	rs16829453	1:188836078	0.02	Add	W	4.2x10^-6^	0.04	6.4x10^-6^
*PLA2G4A|FAM5C*	3	rs16829453	1:188836078	0.02	Dom	W	4.3x10^-6^	0.03	5.5x10^-6^
*MYO16*	1	rs6492128	13:109271217	0.01	Add	W	6.4x10^-6^	0.03	5.7x10^-6^
*MYO16*	1	rs6492128	13:109271217	0.01	Dom	W	6.4x10^-6^	0.03	5.7x10^-6^
*SLC6A11*	1	rs3821754	3:10978825	0.01	Add	W	4x10^-6^	0.04	6.1x10^-6^
*SLC6A11*	1	rs3821754	3:10978825	0.01	Dom	W	4x10^-6^	0.04	6.1x10^-6^
*OLFM4|SUGT1*	3	rs894840	13:53973955	0.09	Add	W	8.7x10^-6^	0.03	8.4x10^-6^
*LOC100289576|GPRC5C*	1	rs8071255	17:72429618	0.01	Add	W	7.2x10^-6^	0.04	1.1x10^-5^
*LOC100289576|GPRC5C*	1	rs8071255	17:72429618	0.01	Dom	W	7.2x10^-6^	0.04	1.1x10^-5^
*SPATA8|LOC91948*	1	rs1500631	15:98079217	0.01	Add	W	7.8x10^-6^	0.05	1.4x10^-5^
*SPATA8|LOC91948*	1	rs1500631	15:98079217	0.01	Dom	W	7.8x10^-6^	0.05	1.4x10^-5^

MAF: Minor allele frequency; Add: additive; Dom: dominant.

^a^B: black, W: white

^b^sex, age and race-specific PC adjusted (4 PCs for whites, 2 PCs for blacks).

^c^meta-analysis combining discovery and validation cohorts, for race-stratified analyses, adjusted for sex, age and race-specific PCs.

In meta-analyses combining the race-stratified results, eleven SNPs were associated with factor 1 (MCA) levels, with three of these SNPs showing more than nominal association (Table 3); one of these (rs10987728 in *CDK9*) was also identified from race-stratified results and two (rs16990949 in PDX1 C-terminal inhibiting factor 1 [*PCIF1*]) and rs543129 [intergenic between cutaneous T-cell lymphoma-associated antigen 1 (*CTAGE1*) and retinoblastoma binding protein 8 (*RBBP8*)]) were new mQTL identified in these race meta-analyses. Eight SNPs were associated with factor 2 (LCDA) levels (Table 3); one gene had been identified in race-stratified analyses (*ZNF521*) but showed stronger association in the validation cohort in these analyses, and rs352216 near frizzled class receptor 3 (*FZD3*) was a new mQTL. Factor 3 (SCDA) again had the largest number and strongest mQTL with fourteen SNPs associated with SCDA levels, with eight SNPs showing more than nominal significance in the validation cohort (Table 3). SNPs in *USP3*, *HERC1* and *OLFM4*|*SUGT1* (intergenic between olfactomedin 4 and SGT1, suppressor of G2 allele of SKP1 [S. cerevisiae]) had already been identified in race-stratified analyses; additional mQTL identified in these race meta-analyses included rs12589750 and rs3853422 (in or near stonin 2 [*STON2*] and sel-1 suppressor of lin-12-like (C. elegans) [SEL1L]), rs930491 and rs11827377 (both intergenic between ribonucleotide reductase M1 [*RRM1*] and stromal interaction molecule 1 [*STIM1*]), rs11242866 (between solute carrier family 22 (organic cation transporter), member 3 [*SLC22A23*] and PX domain containing 1 [*PXDC1*]), and rs4544127 (near FRAS1-related extracellular matrix protein 2 [*FREM2*] and stomatin-like protein 3 [*STOML3*]).

**Table 3:** Significant mQTL for GWAS of metabolite factors, race meta-analyses. Presented are SNPs meeting genomewide significance for association with factor 1 (MCA metabolites), factor 2 (LCDA metabolites) and factor 3 (SCDA metabolites) in race-combined meta-analyses in the discovery cohort (p≤10^−6^) also showing nominal association (p≤0.05) in the validation cohort, ranked by meta-analysis p-value.

Gene	Factor	SNP	Chr:Position	MAF W^a^	MAF B^a^	MAF O^a^	Model	Disc p^b^	Valid p^b^	Meta p^c^
*STON2*	3	rs12589750	14:81891157	0.001	0.10	0.02	Add	2.0x10^-6^	7.7x10^-7^	7.2x10^-12^
*STON2*	3	rs12589750	14:81891157	0.001	0.10	0.02	Dom	1.5x10^-6^	3.5x10^-5^	3.3x10^-10^
*SULF2|PREX1*	3	rs1886848	20:46695252	0	0.05	0.01	Add	9.9x10^-12^	0.02	1.2x10^-10^
*ZNF521*	2	rs12965721	18:22648924	0.16	0.05	0.14	Add	8.5x10^-6^	1.7x10^-5^	7.7x10^-10^
*RRM1|STIM1*	3	rs930491	11:4199848	0.0004	0.07	0.02	Add	3.1x10^-8^	2.0x10^-3^	2.2x10^-9^
*RRM1|STIM1*	3	rs930491	11:4199848	0.0004	0.07	0.02	Dom	6.8x10^-6^	8.2x10^-4^	4.1x10^-8^
*RRM1|STIM1*	3	rs11827377	11:4200685	0.0004	0.07	0.02	Add	3.1x10^-8^	2.4x10^-3^	2.7x10^-9^
*RRM1|STIM1*	3	rs11827377	11:4200685	0.0004	0.07	0.02	Dom	3.1x10^-8^	1.0x10^-3^	5.2x10^-8^
*USP3*	3	rs10450989	15:63846508	0.05	0.004	0.02	Add	5.0x10^-6^	4.9x10^-5^	1.5x10^-9^
*USP3*	3	rs10450989	15:63846508	0.05	0.004	0.02	Dom	6.9x10^-6^	1.1x10^-3^	7.0x10^-8^
*HERC1*	3	rs2228513	15:63950887	0.05	0.004	0.02	Add	5.0x10^-6^	1.3x10^-4^	4.5x10^-9^
*HERC1*	3	rs2228513	15:63950887	0.05	0.004	0.02	Dom	6.8x10^-6^	2.4x10^-3^	1.9x10^-7^
*ZNF521*	2	rs4800615	18:22622445	0.12	0.03	0.12	Add	1.5x10^-7^	3.1x10^-4^	8.8x10^-10^
*ZNF521*	2	rs4800615	18:22622445	0.12	0.03	0.12	Dom	1.3x10^-6^	8.0x10^-4^	1.4x10^-8^
*LIN7A*	1	rs11114645	12:81280092	0.02	0.08	0.04	Add	3.6x10^-9^	0.01	8.9x10^-9^
*RRM1|STIM1*	3	rs11826962	11:4200923	0.0002	0.05	0.01	Add	2.4x10^-8^	0.02	4.7x10^-8^
*RRM1|STIM1*	3	rs11826962	11:4200923	0.0002	0.05	0.01	Dom	5.5x10^-6^	0.02	1.8x10^-6^
*LIN7A*	1	rs12304000	12:81282585	0.02	0.14	0.04	Add	1.1x10^-8^	0.02	5.3x10^-8^
*CDK9*	1	rs10987728	9:130553040	0.01	0.00	0.01	Add	5.6x10^-6^	1.2x10^-3^	7.4x10^-8^
*CDK9*	1	rs10987728	9:130553040	0.01	0.00	0.01	Dom	5.6x10^-6^	9.3x10^-4^	5.7x10^-8^
*OLFM4|SUGT1*	3	rs9591507	13:53929144	0.05	0.12	0.04	Add	4.3x10^-7^	6.4x10^-3^	1.0x10^-7^
*SLC22A23|PXDC1*	3	rs11242866	6:3593956	0.001	0.06	0.02	Dom	3.0x10^-6^	3.6x10^-3^	1.3x10^-7^
*CADM2|VGLL3*	3	rs6796873	3:86168194	0.002	0.23	0.05	Add	1.8x10^-6^	0.01	3.7x10^-7^
*AFAP1L2*	1	rs493347	10:116133734	0.009	0.28	0.08	Dom	6.7x10^-6^	0.02	5.0x10^-7^
*STON2|SEL1L*	3	rs3853422	14:81900169	0.001	0.04	0.0009	Add	7.6x10^-6^	0.01	6.3x10^-7^
*STON2|SEL1L*	3	rs3853422	14:81900169	0.001	0.04	0.0009	Dom	7.6x10^-6^	0.02	1.6x10^-6^
*RAMP1*	3	rs3769047	2:238769892	0.003	0.02	0.04	Add	6.8x10^-6^	0.04	6.4x10^-6^
*RAMP1*	3	rs3769047	2:238769892	0.003	0.02	0.04	Dom	2x10^-7^	0.04	6.6x10^-7^
*FZD3*	2	rs352216	8:28426891	0.001	0.08	0.02	Add	9.4x10^-6^	0.01	1.2x10^-6^
*FZD3*	2	rs352216	8:28426891	0.001	0.08	0.02	Dom	6.1x10^-6^	4.2x10^-3^	2.8x10^-7^
*PCIF1*	1	rs16990949	20:44575493	0.04	0.07	0.03	Add	5.6x10^-6^	0.01	1.3x10^-6^
*PCIF1*	1	rs16990949	20:44575493	0.04	0.07	0.03	Dom	3.6x10^-6^	7.3x10^-3^	5.1x10^-7^
*FREM2|STOML3*	3	rs4544127	13:39538666	0.006	0.12	0.03	Dom	6.5x10^-6^	8.7x10^-3^	7.3x10^-7^
*CTAGE1|RBBP8*	1	rs543129	18:20057092	0.02	0.20	0.11	Add	7.1x10^-6^	4.9x10^-3^	4.8x10^-7^
*CTAGE1|RBBP8*	1	rs543129	18:20057092	0.02	0.20	0.11	Dom	5.8x10^-6^	0.02	2x10^-6^
*FLJ40606|PCIF1*	1	rs8114598	20:44562900	0.04	0.07	0.03	Add	5.7x10^-6^	0.02	2.9x10^-6^
*FLJ40606|PCIF1*	1	rs8114598	20:44562900	0.04	0.07	0.03	Dom	3.6x10^-6^	0.01	1.2x10^-6^
*PLTP|FLJ40606*	1	rs16990934	20:44553194	0.04	0.07	0.03	Add	1.7x10^-6^	0.02	1.2x10^-6^
*PLTP|FLJ40606*	1	rs16990934	20:44553194	0.04	0.07	0.03	Dom	1.0x10^-6^	0.01	4.7x10^-7^
*GATA3|SFTA1P*	1	rs7908673	10:8786636	0.13	0.41	0.14	Add	2.1x10^-6^	0.03	2.3x10^-6^
*LOC151121|LOC389033*	2	rs2165440	2:130007996	0.03	0.13	0.09	Add	4.6x10^-7^	0.03	1.2x10^-6^
*C14orf105*	3	rs10139566	14:57960474	0.01	0.14	0.05	Dom	6.3x10^-6^	0.02	2.2x10^-6^
*GATA3|SFTA1P*	1	rs7908673	10:8786636	0.13	0.41	0.14	Add	2.1x10^-6^	0.03	2.3x10^-6^
*NFIA|TM2D1*	2	rs17122575	1:62104766	0.003	0.06	0.02	Add	2.9x10^-7^	0.05	5.1x10^-6^
*ZFAT|LOC286094*	2	rs7820325	8:135969632	0.30	0.22	0.30	Add	6.9x10^-6^	0.03	6.0x10^-6^
*SLC35B1*	1	rs8186	17:47778793	0.04	0.15	0.05	Add	5.1x10^-6^	0.04	8.2x10^-6^
*LOC100289139|LRRC4C*	2	rs12270585	11:39882450	0.0004	0.10	0.02	Dom	7.0x10^-6^	0.05	0.13
*C18orf20|CDH7*	2	rs1787927	18:62587346	0.002	0.10	0.05	Add	1.1x10^-6^	0.02	0.16

MAF: minor allele frequency; Add: additive model; Dom: dominant model.

^a^W: white, B: black, O: other race

^b^race-stratified results sex, age and race-appropriate PC adjusted, and then combined using meta-analysis.

^c^meta-analysis combining discovery and validation cohorts, race-stratified results combined.

Thus, to summarize, the most robust results overall were for mQTL associated with SCDA metabolite levels (factor 3) including an mQTL composed of *USP3* (rs10450989) and *HERC1* (rs2228513); and a locus composed of *STON2* (rs12589750) and *SEL1L* (rs3853422), with loci meeting genomewide significance in the discovery cohort (p≤10^−6^), strong significance in the validation cohort (p = 2.4x10^-3^–7.7x10^-7^, except rs3853422 which only showed borderline significance [p = 0.01]), and stronger association in the meta-analyses (p = 1.6x10^-6^–7.2x10^-12^). The next strongest overall results for SCDA mQTL (based on race-stratified or race-combined meta-analysis p-values) in descending order of significance were for *RRM1|STIM1*, *OLFM4|SUGT1*, *SLC22A23|PXDC1*, *RSBN1L*, *FBXO25|ERICH1*, and *FREM2*|*STOML3*. The next strongest results overall were for mQTL associated with LCDA (factor 2) levels with SNPs in *PIGR*, *ZNF521*, *USH2A* and *FZD3* showing more than nominal significance in the validation cohort. Finally, mQTL associated with MCA (factor 1) levels included *CDK9*, *DIRC3*, *CTAGE1*|*RBBP8*, and *PCIF1*.

**Fig 2:**
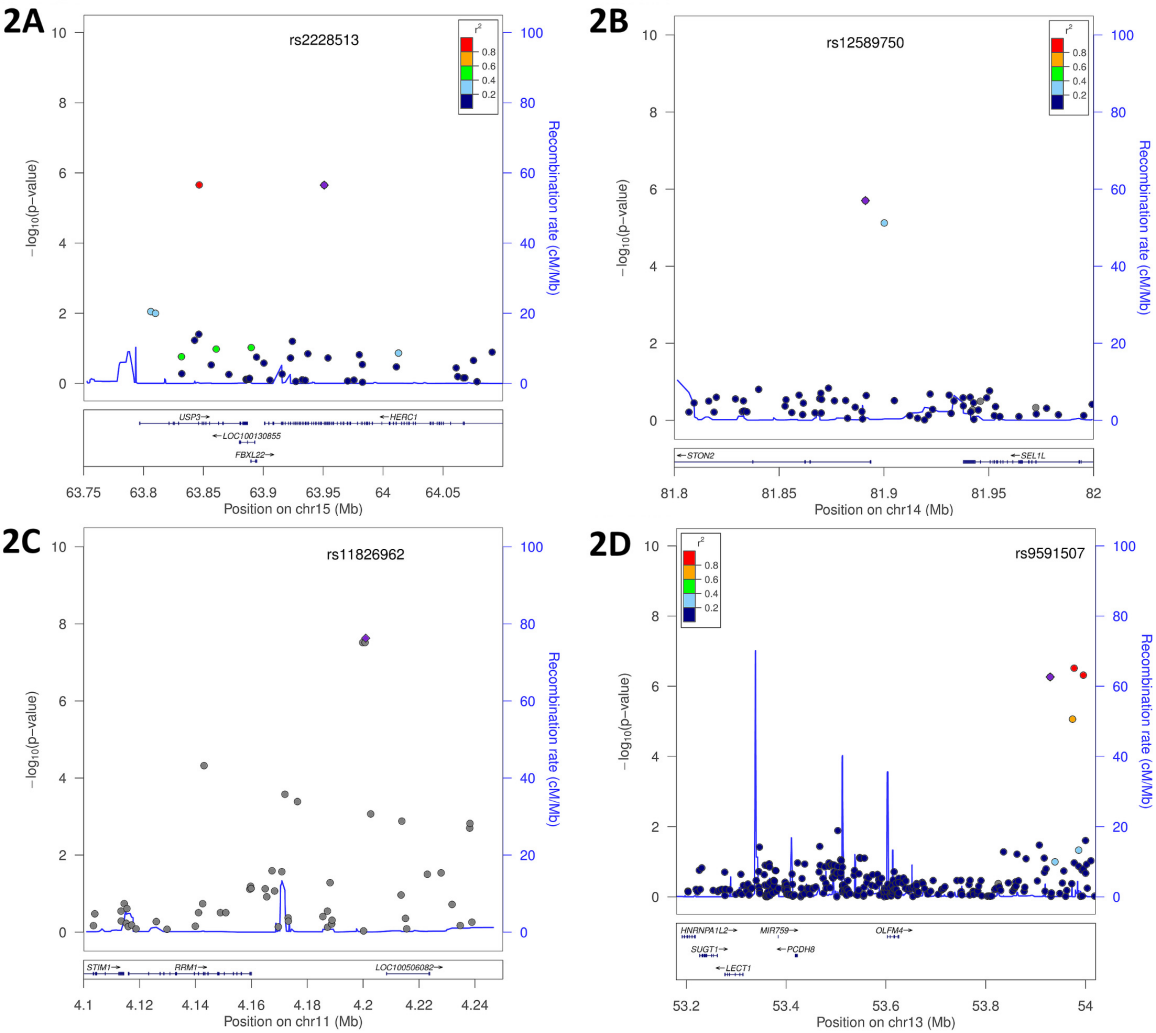
Genomic region plots for significant mQTL associated with SCDA levels. Displayed are LocusZoom plots with -log10(p-value) (left Y-axis) and LD (right Y-axis), additive model, discovery cohort: **(A)** *USP3*|*HERC1*, whites only; **(B)** *STON2|SEL1L*, race meta-analysis; **(C)** *RRM1*|*STIM1*, race meta-analysis; **(D)** *OLFM4*|*SUGT1*, whites only.

We have previously shown that all three metabolite factors predict risk of incident CVD events, however the results from those studies were most robust for the SCDA metabolites [5]. Given these prior results, and the strength and consistency of findings for the SCDA metabolite factor in these GWAS analyses, we chose to focus the remainder of our analyses on this factor. Fig 2 and S5 Fig display Locus Zoom plots for these eight mQTL most strongly associated with SCDA metabolite factor levels. Interestingly, the majority of these (i.e. *HERC1*, *USP3*, *STIM1*, *SUGT1*, *FBXO25* and *SEL1L*) encode proteins reporting on endoplasmic reticulum (ER) stress.

### SCDA mQTL SNPs also associate with incident cardiovascular events

SCDA mQTL were tested for association with incident CVD events using Cox proportional hazards time-to-event analyses in the combined discovery and validation datasets, using meta-analysis of race- and dataset-stratified results, unadjusted for multiple comparisons. Of these eight mQTL (15 SNPs) loci, four SNPs predicted mortality in additive models: *HERC1* rs2228513 (p = 0.05 in race combined, p = 0.04 in whites only), *RRM1* rs11826962 (p = 0.03), and *FBOX025* rs1869075 (p = 2.5x10^-4^ for blacks only, not significant in race combined analyses), with *USP3* rs10450989 showing a trend for association (p = 0.06 in race combined, p = 0.05 in whites only). FREM2|STOML3 rs4544127 showed a trend for association (p = 0.06). We observed for the *HERC1* SNP a 33% event rate for non-carriers and a 36% event rate for carriers of at least one copy of the minor G allele (the same allele associated with higher SCDA levels, S3 Table). Adjustment for SCDA levels in these models resulted in attenuation of the association between mQTL and CVD event (S3 Table), suggesting that the relationship between these mQTL and CVD events is in part mediated through SCDA metabolite levels.

### mQTL SNPs are associated with SCDA independent of risk factors

To ensure that the relationships between SNPs and SCDA levels were not confounded by renal disease, we further adjusted for glomerular filtration rate. This adjustment caused no or minimal attenuation of the association for our strongest SNPs (S3 Table). In multivariable models, we found minimal attenuation of the association between most SNPs and SCDA levels (S3 Table), suggesting that these SNPs have effects on SCDA levels unrelated to other comorbidities. There was attenuation of association of SNPs near *RRM1|STIM1* and *STON2|SEL1L* after adjustment (although still significant at p<0.05, unadjusted for multiple comparisons), suggesting that these SNPs have effects on SCDA levels mediated through these clinical factors, in particular renal disease.

### Methylation in ER stress genes is different in individuals with extremes of SCDA metabolites

Visual comparison of the distribution of methylated probes revealed similar distributions in individuals with high and low SCDA levels (N = 46, combined methylation discovery and validation datasets, S6 Fig). After filtering based on Δβ values, the presence of multiple correlated probes in a gene, and adjustment for estimated cell type proportions, sex, age and race, probes in 28 genes showed differential methylation in SCDA extremes (i.e. |Δβ|≥0.10 in ≥2 probes within a gene). Differential methylation in three of these genes was confirmed in the validation set based on |Δβ|≥0.10 (*BRSK2*, *Hook2* and *LMTK3*, Table 4). Two of these genes, including the most significant one, report on ER stress: *Hook2* (four probes, Δβ 0.25–0.30) and *BRSK2* (four probes, Δβ 0.11–0.20). *Hook2* may be involved in pathways contributing to the ubiquitin proteasome system (UPS) arm of ER stress via its role in establishment and maintenance of pericentrosomal localization of aggresomes (complexes of misfolded proteins, chaperones and proteasomes) [8]. *BRSK2* encodes brain selective kinase 2, a serine/threonine kinase of the AMPK family that acts as a checkpoint kinase in response to DNA damage induced by UV irradiation. BRSK2 protein levels are down-regulated in response to ER stress and ER stress promotes localization of BRSK2 to the ER [9]. Knockdown of endogenous *BRSK2* expression enhances ER stress-mediated apoptosis in human pancreatic carcinoma and HeLa cells [9].

**Table 4:** Whole genome methylation profiling. Genes showing highest degree of differential methylation between individuals with high and low SCDA levels with |Δβ|>0.10 in the discovery and validation datasets.

Gene	Chromosome (bp location)	No. Probes^a^	Δβ^b^	Lowest p-value model 1^c^	Lowest p-value model 2^d^
*BRSK2*	11 (1411129..1483919)	8	0.11–0.20	2x10^-3^	7x10^-4^
*HOOK2*	19 (12873817..12886434)	4	0.25–0.30	6x10^-3^	0.10
*LMTK3*	19 (49000897..49002338)	3	0.10–0.12	0.07	0.20

^a^number of differentially methylated probes within gene

^b^methylated (M) and unmethylated (U) signal intensities and overall methylation levels (β) were calculated as the ratio of methylated to total signal (i.e. β = M / (M + U)) where β ranges from 0 (unmethylated) to 1 (methylated). Δβ was calculated for the difference in overall methylation levels between high and low SCDA level individuals.

^c^nominal p-value, unadjusted for multiple comparisons; adjusted for estimated cell proportions.

^d^nominal p-value, unadjusted for multiple comparisons; adjusted for estimated cell proportions, age, race and sex.

### Expression quantitative trait loci (eQTL) analyses also implicate the ubiquitin proteasome arm of ER stress

Blood RNA microarray data were generated for *N* = 1204 CATHGEN individuals. We began by examining *cis* effects for the identified SNPs; however, many of the top SNPs did not have available *cis*-transcripts after extensive QC. Rs9591507,rs17573278, rs894840, and rs9285184 (all in *OLFM4*|*SUGT1*), rs11771619 (*RSBN1L*), rs1869075 (*FBXO25*), and rs1886848 (*SULF2*) showed evidence of *cis*-regulation (S4 Table). *HERC1* and *USP3* are not well-represented on the microarray (one probe per gene); there was only a minimal trend toward association between the *HERC1* and *USP3* SNPs with *HERC1* expression (p = 0.16 and 0.19, respectively) and no association with the *USP3* transcript.

We then performed eQTL analyses to find evidence of *trans*-acting pathways (S4 Table). When analyzed as single transcripts, among the top ten transcripts associated with *HERC1* rs2228513 and *USP3* rs10450989 were *USP39* (p = 0.0002 and p = 0.0004, respectively) and *CYLD* (p = 0.00015 and p = 0.0007), suggesting that these SNPs show functional relationships with expression of *trans*-acting pathways related to the UPS arm of ER stress. USP39 has a role in pre-mRNA splicing and is essential for recruitment of the U4/U6.U5 tri-snRNP to the prespliceosome. The tumor suppressor CYLD is a deubiquitinating enzyme, acts as a negative regulator of NF-kappa-B signaling, and plays a pro-inflammatory role in vascular smooth muscle cells [10]. Cis- and trans-eQTL analyses were not adjusted for multiple comparisons, as we were looking for focused functional effects for each SNP.

Using GSEA [11], we then identified KEGG pathways of transcripts associated with each SNP; nominal p-values are reported. The most significant pathway associated with *HERC1* rs2228513 was “ubiquitin mediated proteolysis” (p = 0.01; p = 0.12 for *USP3* rs10450989). The most significant pathway for rs10450989 was “RNA degradation” (p = 0.03). Pathways associating with the other SNPs reported on various cellular processes: rs930491 and rs11827377 (*RRM1|STIM1*) with RNA polymerase pathway (both p = 0.001); rs11826962 (*RRM1|STIM1*) with JAK-STAT signaling pathway (p<0.0002); rs17573278 (*OLFM4|SUGT1*) with Alzheimer’s disease pathway (p = 0.008); rs894840 (*OLFM4|SUGT1*) with glycosaminoglycan biosynthesis (p<0.0002); rs12589750 and rs3853422 (*STON2|SEL1L*) with ribosome pathway (p<0.0001 and p = 0.001, respectively) and FC Gamma R mediated phagocytosis pathway (p = 0.001 for both). The Alzheimer’s disease pathway includes components of ER stress and there is evidence that neuronal death in Alzheimer’s disease may arise from ER dysfunction. The ER is also thought to play an important structural role in phagocytosis.

Finally, we performed GSEA for the correlation between SCDA levels with genomewide RNA expression; nominal p-values are reported. The most significant KEGG pathways were oxidative phosphorylation (p<0.0002), Parkinson’s disease (p<0.0002), cardiac muscle contraction (p<0.0002), porphyrin and chlorophyll metabolism (p = 0.002), and the proteasome pathway (p = 0.008). The proteasome is an integral component of the UPS arm of ER stress, degrading cellular proteins that are modified by ubiquitin. Also, an integral part of the Parkinson’s disease pathway includes components of the UPS.

### Biochemical characterization of SCDA metabolites

In this and prior studies [4–6], SCDA were measured using a flow-injection-MS/MS method that is ideal for rapid profiling of samples, but full resolution of isomeric species comprising each SCDA metabolite peak is not achieved. C6-DC represents a SCDA that loads heavily on the PCA-derived SCDA factor in our studies, which can be comprised of either the branched-chain methylglutaryl acylcarnitine or the straight chain adipoyl acylcarnitine isomers. To resolve these metabolites, we adapted a liquid chromatography (LC)-MS/MS method [12]. Peak identification was facilitated by in-house chemical synthesis of internal standards for the two targeted analytes [13]. Using this method, we re-analyzed 29 human plasma samples from our original studies [5] that contained the highest C6-DC levels. We found that in the majority of individuals (19 of 29), the clearly predominant C6-DC isomer was the branched-chain 3-methylglutaryl carnitine metabolite, and in in 23 of the 29 individuals levels of the branched chain isomer were higher than the straight chain isomer (S7 Fig). The correlation between the C6-DC measured by flow injection-MS/MS with each of these LC-MS/MS measured isomers further confirms that it is primarily the branched-chain isomer accounting for the signal (r2 = -0.06, p = 0.8 for straight chain isomer; r2 = 0.67, p = 1.8x10^-4^ for branched-chain isomer). Interestingly, one potential source of the branched-chain 3-methylglutaryl carnitine metabolite is the branched-chain amino acid leucine. Our previous studies have shown an association of branched-chain amino acid metabolites with coronary artery disease [4, 7].

### ER stress markers increase in conjunction with SCDA metabolites in cultured cells

**Fig 3:**
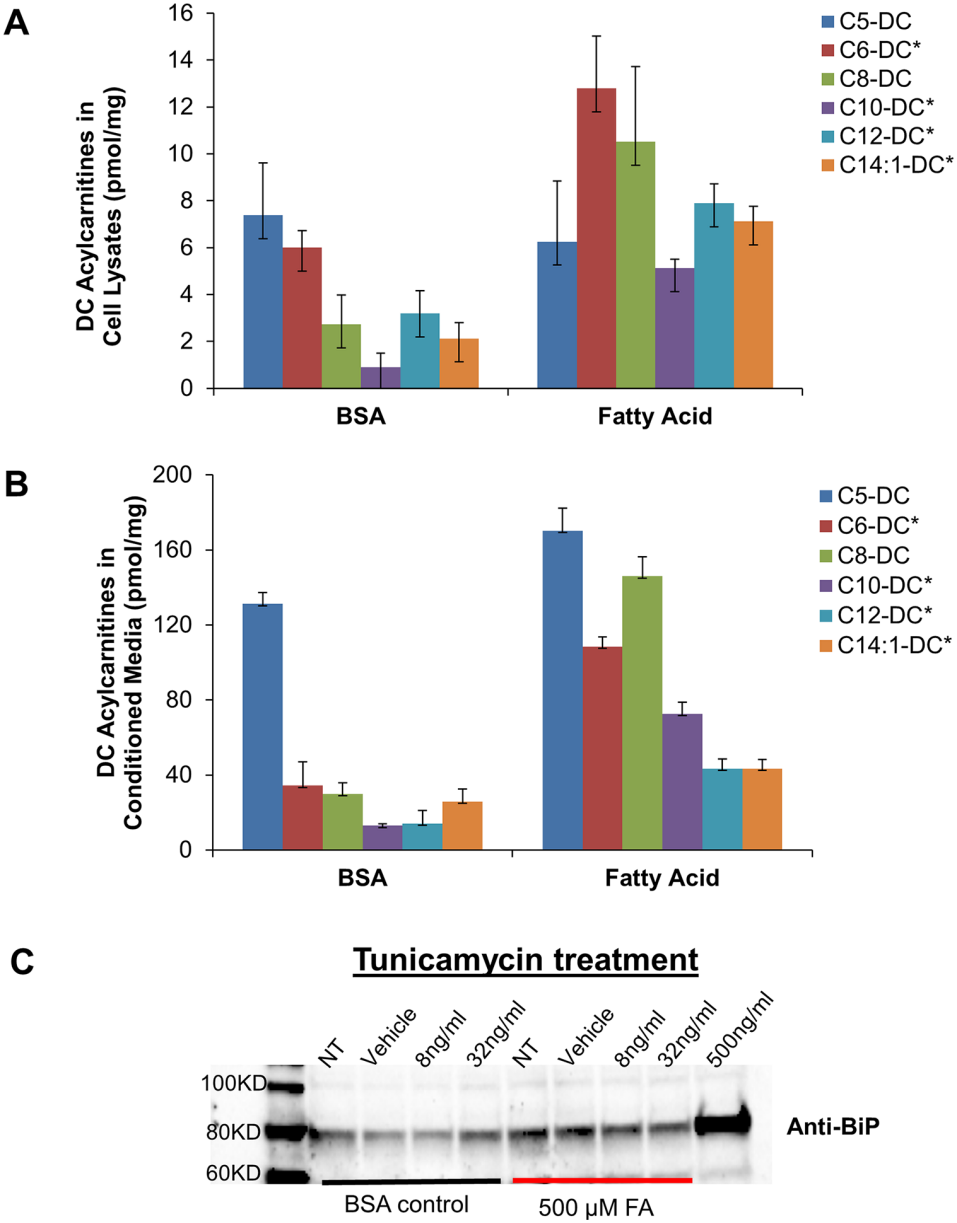
Dicarboxylic (DC) acylcarnitines measured in HEK 293 cell lysates (A) and conditioned medium (B) after 24 h exposure to BSA alone or in complex with 500 uM fatty acids (FA, oleate:palmitate, 1:1). C) Representative Western blot analysis of the ER stress protein, BiP, in HEK 293 cells treated 24 h with 500 uM FA ± increasing doses of tunicamycin (NT; no treatment, Vehicle (DMSO), 8 ng/mL and 32 ng/mL tunicamycin). High dose tunicamycin (500 ng/mL) served as a positive control. Asterisks indicate significant difference between BSA and FA experiments (p<0.05).

The above findings linking ER stress to SCDA metabolites led us to question whether nutrient-induced accumulation of dicarboxylacylcarnitines would be accompanied by ER stress in cultured cells. Exposure of human HEK293 kidney cells to 500 uM fatty acids for 24 hours (a condition designed to mimic elevated fatty acid levels observed in human obesity) increased cellular production and efflux of several long, medium and short-chain dicarboxylacylcarnitines (Fig 3A and 3B). Interestingly, fatty acid-induced production of dicarboxylacylcarnitines was accompanied by elevated expression of the molecular chaperone protein BiP (Fig 3C), a well-recognized marker of ER stress. At low doses of the ER stress agent tunicamycin (lower than required to cause cytotoxicity), fatty acid exposure also augmented BiP expression (Fig 3C). Together, these results point to an intriguing connection between cellular carbon load, dicarboxylic acylcarnitines and proteotoxicity.

## Discussion

We have analyzed metabolomics, genetics, epigenetics and transcriptomics together to establish genomewide associations between a cluster of SCDA metabolites that predict CVD events and specific genetic loci. Our findings implicate the UPS arm of ER stress as a factor influencing SCDA levels and CVD event pathogenesis. Several previous studies have successfully mapped metabolites to genetic loci [2], but primarily have not triangulated such genetic variation with disease endpoints and functional studies. Key findings of the current study include: (1) SNPs and CpG probes in genes reporting on components of ER stress were associated with levels of SCDA metabolites previously shown to predict CVD events [3–5]; (2) several of these SNPs themselves also predicted CVD events; (3) some of the SNPs/genes were linked with SCDA metabolites and ER stress through eQTL analyses; (4) the isomeric composition of the peak containing the major SCDA metabolite C6-DC was clarified; and (5) in cultured cells, nutrient-induced accumulation of SCDA metabolites occurred in parallel with increases in the ER stress marker BiP. Subjects in the CATHGEN cohort have a high prevalence of obesity, hyperlipidemia and diabetes (Table 1). Thus, our *in vitro* experiment may be viewed as a mimetic of the metabolic environment to which CATHGEN subjects are commonly exposed.

Our strongest finding was for two SNPs (*HERC1* rs2228513 and *USP3* rs10450989) that are in LD (r2 = 0.99) despite being separated by 104 kB. Rs2228513 is a missense variant (serine to phenylalanine) that is predicted to be “probably damaging” by PolyPhen, but no functional evaluation has been reported. Rs10450989 is an intronic SNP. The HERC gene family encodes a group of large proteins that contain multiple structural domains including a C-terminal HECT domain found in a number of E3 ubiquitin protein ligases. HERC1 is involved in membrane trafficking and may also act as an E3 ubiquitin-protein ligase, a protein that accepts ubiquitin from an E2 ubiquitin-conjugating enzyme and then directly transfers the ubiquitin to targeted substrates. Rs2228513 corresponds to residue 3152, which does not map to a specific domain in the protein. Our eQTL results suggest that this SNP is associated with differential expression of genes within a pathway reporting on the UPS. *USP3* encodes ubiquitin-specific protease 3 which mediates release of ubiquitin from degraded proteins by disassembly of the polyubiquitin chains in the ER. Deubiquitination has been implicated in cell cycle regulation, proteasome-dependent protein degradation, and DNA repair [14]. Interestingly, an intergenic SNP 58 kB upstream from *USP3* (rs10519210) was the strongest SNP associated with heart failure in a GWAS from the CHARGE consortium [15]. Rs10519210 not associated with SCDA levels in our study (p = 0.16) and is not in LD with rs10450989 (r2 = 0.002). Our next strongest finding was for a locus in/near *STON2* and *SEL1L*. Rs12589750 is an intronic SNP within *STON2* and rs3853422 is intergenic between *STON2* and *SEL1L*. SEL1L plays a role in the ER-associated protein degradation (ERAD) machinery, and is part of a complex necessary for the retrotranslocation of misfolded proteins from the ER lumen to the cytosol where they are then degraded by the proteasome in a ubiquitin-dependent manner. Dysfunctional protein degradation causes ER stress.

Other mQTL included SNPs near *RRM1* and *STIM1*; *STIM1* encodes a calcium sensor in the ER that translocates to the plasma membrane upon calcium store depletion to activate calcium release-activated calcium channels. STIM1 induction, redistribution and clustering are important during ER stress when calcium stores are depleted [16]. *FBXO25* is one of 68 human F-box proteins that serve as specificity factors for a complex composed of s-phase-kinase associated protein 1 (Skp1) and cullin1 (SCF), that act as protein-ubiquitin ligases, targeting proteins for destruction across the UPS. *FBXO25* is cardiac specific and acts as a ubiquitin E3 ligase for cardiac transcription factors [17]. Rs17573278 and rs9591507 are intergenic SNPs >400 kB downstream from *OLFM4* and *SUGT1*. *SUGT1* is required cell cycle transitions and encodes a novel subunit of the SCF ubiquitin ligase complex [18]. *OLFM4* encodes an anti-apoptotic protein that promotes tumor growth. The functions of the other SCDA mQTL loci are unclear.

Given the strength of association of SCDA metabolites (factor 3) with CVD and their particular strength of association in the current GWAS analyses, we chose to focus our subsequent analyses on SCDA. However, we did also identify mQTL for LCDA and MCA, both of which have also been shown to predict CVD events. LCDA are metabolic intermediates of long chain fatty acid oxidation in the mitochondria or peroxisomes. The most significant mQTL for LCDA metabolite levels included *PIGR*, *USH2a*, *ZNF521* and *FZD3*. *PIGR* is a member of the immunoglobulin superfamily and *ZNF521* is involved in regulation of early B-cell factor, suggesting a potential relationship between LCDA levels and immune and/or inflammatory pathways as a link to CVD. MCA are byproducts of mitochondrial fatty acid oxidation. The most significant mQTL for MCA show no obvious potential biologic relationship to mitochondrial function and/or CVD. More epidemiologic and functional work is necessary to clarify these links.

Importantly, and unique to this study, we have observed an association of mQTL and disease phenotypes. The SNPs most significantly associated with SCDA levels (*HERC1* and *USP3*) were also associated with CVD events, with a consistent direction of effect (G allele associating with higher SCDA levels and events). *STIM1*|*SEL1L* SNPS were not associated with CVD events despite their strong association with SCDA levels; this may be due to limited power related to the low MAF in racial subsets. Adjustment for SCDA levels in these models resulted in attenuation of the association between SNP and CVD event suggesting that the relationship between underlying mQTL and CVD events is in part or in full mediated through SCDA metabolites and not through a different biological pathway. In combination, these results suggest potential functional and pathway relationships between SCDA metabolites and CVD events.

We also integrated transcriptomics and whole genome methylation with SNP and metabolomic data sets. eQTL identified ER stress pathways, and specifically those reporting on the ubiquitin proteasome pathway, as associated with the SNPs linked to SCDA via GWAS, and with SCDA metabolites themselves. Whole genome methylation identified epigenetic regulation of genes in ER stress pathways to be associated with extreme SCDA levels. These results support the concept that these polymorphisms and ER stress underlie the relation between SCDA metabolites and CVD events. Finally, we clarified the biochemical structure of the metabolite most strongly accounting for the C6-DC SCDA peak; these results will enable more accurate identification of the source pathways for C6-DC and other SCDA in future studies.

**Fig 4:**
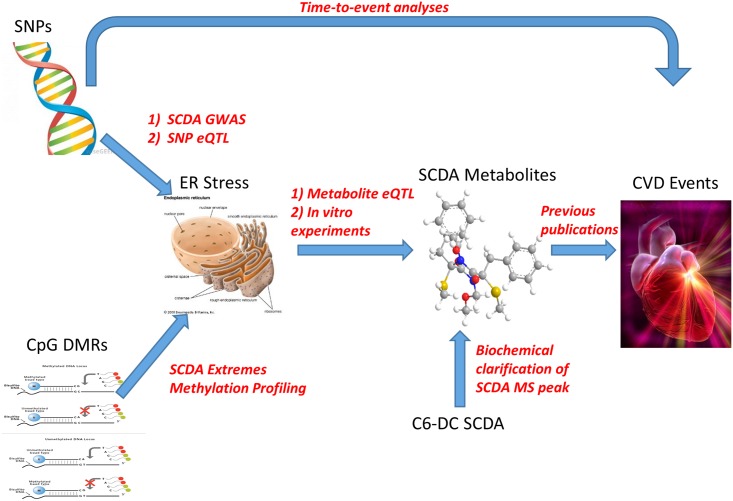
Representation of metabolomics, GWAS, eQTL, and methylation leading to convergence on ER stress as a pathway for CVD event pathogenesis.

Many SCDAs result from the catabolism of amino acids, ω-oxidation of fatty acids or perhaps represent products of microbial metabolism [19], but the reasons for their accumulation in plasma in at-risk subjects, and how they may be related to CVD pathogenesis remain uncertain. Based on the convergence of GWAS, transcriptomic, metabolomic and functional data presented herein, we hypothesize that genetic and epigenetic variation predisposes to increased susceptibility to ER stress through proteasome dysfunction (reflected by the observation of upregulation of expression of ER stress genes), with ER stress in turn contributing to increased production of SCDA metabolites. This pathway of increased ER stress then leads to increased risk of CVD events, with SCDA metabolites and the genetic variants themselves predicting increased risk by reporting on this pathway (Fig 4). Epigenetic variation could be the influence of environmental or lifestyle factors inducing methylation changes; in this working model, diet and lifestyle-induced dyslipidemia and hyperglycemia could result in methylation changes as a regulatory mechanism to handle nutrient overload, thus predisposing to dysregulated ER stress which then leads to subsequent CVD events.

The UPS arm of the ER is responsible for the removal of misfolded proteins but is sometimes insufficient, for example, in the setting of increased production of misfolded proteins. The associated proteasome functional insufficiency can lead to cellular dysfunction and cell death, with cardiomyocytes being particularly vulnerable due to limited regenerative capability [20]. The UPS has been hypothesized to be involved in atherosclerosis based on the recognized roles of inflammation, oxidative stress, and endothelial dysfunction in this condition, and the intertwined relationships between the UPS and those pathways [21]. Preclinical evidence of the role of the UPS in atherosclerosis includes studies showing that oxidized LDL inhibits proteasomal activity in macrophages leading to apoptosis [22], and data suggesting that the UPS may contribute to foam cell formation by suppression of apoptosis of lipid-bearing macrophages by aggregated LDL in *in vitro* models [23]. Studies of proteasome inhibition have shown conflicting data; Hermann *et al*. found aggravation of atherosclerosis [24] and myocardial dysfunction [25] in pigs treated with proteasome inhibition, whereas a recent study showed reversal of uremia-induced atherosclerosis with proteasome inhibition in rabbits [26].

Human studies suggesting the role of the UPS in atherosclerosis are limited. Very small studies have shown greater amounts of ubiquitin conjugates in carotid endarterectomy tissues with unstable as compared with stable plaque morphologies [27] and increased UPS activity in carotid tissue from patients with symptomatic compared with asymptomatic carotid disease [28]. While preclinical studies have suggested the role of UPS in atherosclerosis as secondary to oxidative stress or other pathophysiologies, our identification of genetic variants in UPS/ER stress genes using unbiased analyses in our human cohorts provides strong support for the direct etiologic role of the UPS in promoting long-term cardiovascular risk. Importantly, we note that while ER stress is a common pathway in several disorders, we believe that the convergence of results on the UPS highlights its unique relationship to SCDA metabolism.

Our findings could have significant translational implications beyond CVD. Our primary objective of discovery of novel genetic risk variants using an mQTL approach was successful; the unexpected finding of genetic variation predisposing to ER stress could have much broader importance to human disease. Indeed, the response to ER stress is a trait that is known to be heritable in humans [29], but the genetic architecture has not been characterized. Equally as important, our data suggest the presence of easily quantifiable circulating biomarkers of ER stress, traditionally measureable only in tissue through ER stress-responsive gene expression studies. Thus, these results could have more wide-reaching implications for ER stress research in humans. Our prior work solidified the role of SCDA metabolites as predictors of CVD events [4, 5]; the current study has implications for clinical translation using SCDA metabolites for improved risk stratification even beyond CVD given the central role of normal and dysfunctional ER stress in health and disease.

The strengths of this study are the use of *a priori* defined discovery and validation cohorts; integration of genetics, epigenetics, metabolomics, transcriptomics in large cohorts; and careful biochemical refinement of the most strongly associated SCDA metabolite. Importantly, this represents one of the first studies to successfully identify genetic variants through mapping of intermediate metabolomic traits that themselves associate with disease endpoints. Our prior work had consistently identified SCDA metabolites as incremental predictors of CVD events, but little was known about the biological pathways underlying that association; the genomewide, multiple platform molecular approach taken in our study facilitated identification of the UPS more rapidly than other scientific methods. This work also adds an important finding to the metabolomics literature, namely that SCDA metabolites may be reporting on increased or dysregulated ER stress and specifically to proteasome functional insufficiency or dysregulation.

There are limitations to the study; the study population was comprised of individuals referred due to a suspicion of CVD and thus represents a disease-prone population. However, we note that 44% of study participants did not have significant coronary artery disease, highlighting the importance of the detailed angiographic phenotype to ensure that coronary artery disease is not confounding the relationship between genetic factors and outcome. Further, the high burden of CVD risk factors mirrors that of the general population, enabling generalizability of the study findings. Some of the results were isolated to a racial subset because the identified SNPs were either monomorphic or extremely rare in other races, underscoring the potential importance of including non-Caucasian races in such studies. Race-stratified sequencing of these genomic regions may identify different variants in these genes present in other races that may also serve as SCDA and CVD genetic variants. We *a priori* chose a p-value ≤10^−6^ as genomewide significant based on the commonly used threshold at the time we embarked on this study, and as a balance between the overly conservative Bonferroni correction and presence of linkage disequilibrium across the genome. More contemporary GWAS platforms cover a greater number of SNPs and include imputed SNPs in analysis, thus p<10^−8^ is now often considered genomewide significant; most of the key SNPs in this study would meet that threshold in combined meta-analyses, but not in the discovery cohort alone. The significance level also did not account for testing of two genetic models and for race-stratified analyses, however, most of the identified mQTLs would remain significant even after accounting for such multiple testing (p<3.0x10^-7^). More importantly, the use of a validation cohort and convergence of diverse omic’ data on the UPS obviate concerns about type I error with the threshold used for this study. Finally, while our study overall analyzed metabolomics with genetics, epigenetics and transcriptomics, not all individuals were profiled with all platforms, such that we co-analyzed genetic, epigenetic and transcriptomic data with metabolomics data one pair at a time. The ultimate goal for an eventual true systems biology approach would integrate all molecular platforms to unravel molecular pathways. However, to our knowledge this is the largest study deploying four diverse platforms in conjunction with cardiovascular event outcomes to date, and our consistent findings across platforms support further mechanistic interrogation of the identified pathway.

Our results highlight the power of combined molecular analyses and mapping of intermediate disease-related biomarkers for identifying the genetic architecture underlying common complex diseases, and could lead to improved CVD event risk prediction models as well as further mechanistic investigations of the role of the ubiquitin proteasome system in CVD.

## Materials and Methods

### Study design

The overall objective of this study was to integrate metabolomic, genetic (genomewide association study [GWAS]), transcriptomics and epigenetic data in a large human cohort to identify the genetic architecture regulating metabolite levels (metabolites shown to be incrementally predictive of CVD events [4, 5]) and thereby identify novel CVD risk genes. The analytic process was as follows (S1 Fig): (1) a GWAS was conducted of metabolite factor levels in a discovery cohort (N = 1490) individuals from the CATHGEN biorepository; (2) SNPs meeting genomewide significance from the discovery cohort were validated in a second cohort (N = 2022) CATHGEN subjects; (3) to identify potential epigenetic variation regulating SCDA metabolite levels (factor 3), analyses of whole genome methylation profiling of CATHGEN individuals with extremes of SCDA metabolite levels was performed (N = 46); (4) to elucidate potential downstream biological pathways, these validated GWAS SNPs were then tested for association using genomewide transcriptomic data (i.e., eQTL, N = 1204 CATHGEN individuals); similar analyses were conducted using SCDA metabolite levels and transcriptomic data. These analyses identified the UPS arm of ER stress and functional *in vitro* studies of that pathway were then conducted.

### Study population

Individuals were selected from the CATHGEN biorepository of patients referred for evaluation of ischemic heart disease recruited sequentially through the cardiac catheterization laboratories at Duke University (Durham, NC) [30]. After informed consent, blood was obtained from the femoral artery, immediately processed to separate plasma, and frozen at -80°C. Individuals were fasting for a minimum of six hours prior to collection. Patients with severe pulmonary hypertension or transplant were excluded. The discovery cohort for mQTL GWAS analysis of metabolite levels consisted of a coronary artery disease (CAD) case-control sample; CAD cases were defined as having one to three coronary arteries with clinically significant stenosis (i.e. >50%). Controls were defined as not having clinically significant CAD (i.e. zero coronary arteries with >50% stenosis) and being free of cardiovascular disease, peripheral vascular disease and with a normal ejection fraction (LVEF>40%), and were matched to cases on age, race and sex (745 cases and 745 matched controls). This CAD definition was also used as a covariable in multivariable models assessing the association between mQTL and metabolite levels. To ensure generalizability of the mQTL results, the validation cohort for the metabolite GWAS consisted of a sequential cohort of 2022 CATHGEN individuals [30], and was not constrained on CAD or other status. Significant mQTL were tested for association with incident CVD events (death at any time during follow-up). All CATHGEN participants provided informed, written consent for participation in the CATHGEN biorepository at the time of enrollment. The Duke Institutional Review Board (IRB) approved the CATHGEN biorepository and this substudy.

### GWAS genotyping

The Illumina Human Omni1-Quad Infinium Bead Chip (Illumina, San Diego, CA, USA) was used for genotyping in both the discovery and validation cohorts following the manufacturer’s protocol using 200 nanograms of DNA. Quantification of DNA samples prior to genotyping was performed using the Quant-iT PicoGreen dsDNA reagent in a 96-well plate format (Life Technologies, Grand Island, NY, USA). DNA quality was assessed using gel electrophoresis. All samples were scored on a zero to five scale and samples with a score <3 were not further used. Briefly, the samples were denatured and amplified overnight, followed by fragmentation, precipitation and resuspension. DNA was then hybridized to the Illumina BeadChip for 16–24 hours, washed to remove unhybridized DNA, and then labeled with nucleotides to extend the primers to the DNA sample. After the genotyping protocol, BeadChips were imaged using the Illumina iScan system. Genotypes were called using Illumina’s GenomeStudio V2010.2 software (version 1.7.4 Genotyping module). Any SNPs with <98% call frequency, minor allele frequency (MAF)<0.01 in all races, or out of Hardy-Weinberg equilibrium (p<10^−6^) were excluded, resulting in the following number of autosomal SNPs for analysis: 785,945 in whites; 881,891 in blacks; and 871,209 in the “other” race (primarily Native American). Samples with <98% call rates for all SNPs, gender mismatches, cryptic relatedness, or with outlying ethnicity (as determined by multidimensional scaling plots of a linkage disequilibrium-pruned set of SNPs) were excluded (172 samples).

### Metabolomic profiling

Quantitative determination of levels of 63 metabolites (45 acylcarnitines, 15 amino acids, total ketones, β-hydroxybutyrate, and total non-esterified fatty acids [NEFA]) was performed in N = 3512 individuals from the CATHGEN study (N = 1490 for discovery cohort, N = 2022 for validation cohort), using methods as we have done previously [4–6]. Ketones (total and β-hydroxybutyrate) and NEFA were measured on a Beckman-Coulter DxC600 clinical chemistry analyzer, using reagents from Wako (Richmond, VA). For MS-profiled metabolites (acylcarnitines, amino acids), proteins were first removed by precipitation with methanol. Aliquoted supernatants were dried, and then esterified with hot, acidic methanol (acylcarnitines) or *n*-butanol (amino acids). Analysis was done using tandem flow injection MS with a Quattro Micro instrument (Waters Corporation, Milford, MA). Quantification of the “targeted” intermediary metabolites was facilitated by addition of mixtures of known quantities of stable-isotope internal standards. Given the use of internal standards permitting absolute quantification of the metabolites in micromolar concentrations, values below the lower limits of quantification (LOQ) were reported and analyzed as “0”. Metabolites with >25% of values below LOQ were not analyzed (two acylcarnitines: C6 and C7-DC).

### RNA microarray

RNA purification processing was done utilizing Qiagen PAXgene Blood RNA MDx Kits in frozen whole blood PAXgene tubes. Strict adherence to the PAXgene Blood RNA MDx Kit Handbook, Second Edition, July 2005 protocol was maintained throughout the purification process. The purification process failed on 384 samples (four batches of ninety-six samples each) during processing for unidentified reasons and the samples were not repeated. Biotinylated total RNA was generated using the Illumina TotalPrep RNA amplification kit (Life Technologies, Grand Island, NY, USA); 200 nanograms of RNA was used for the kit. The quality of the RNA was determined using the Bioanalyzer RNA Nano chip assay (Agilent, Santa Clara, CA, USA). Quantification of the RNA was determined using the Quant-iT RiboGreen RNA Assay Kit. Samples with RIN scores less than 6.0 were not carried forward. The Human HT-12v3 Expression BeadChip (Illumina, San Diego, CA) was used for quantitative RNA profiling and scanned on the Illumina iScan system according to manufacturer’s protocol. Biotinylated RNA (750 nanograms) was hybridized to the BeadChip and washed; Cy3-SA was then introduced to the hybridized samples and the BeadChips scanned on the Illumina iScan system according to manufacturer’s protocol. Quality control (QC) and background subtraction was performed using Illumina GenomeStudio tools. Probes with a detection p-value <0.05 and detected in >50% of samples were retained for analysis. Expression values were log2 transformed and quantile normalized using Robust Multichip Average (RMA) methods. Results were visually inspected for outliers and sample failures after plotting for variance components comprising eight distinct and standard QC variables at the plate, chip and individual level. A total of 12,800 probes passed the detection and QC filters and 1204 samples passed the QC and outlier filters.

### Statistical methods

Principal components analysis (PCA) with varimax rotation was used for data reduction of metabolomic data from the combined cohorts (S1 Table and S2 Table) using SAS v9.1 (Cary, NC). Factor 1 (composed of a cluster of medium-chain acylcarnitines [MCA]), factor 2 (composed of a cluster of long-chain dicarboxylacylcarnitines [LCDA]), and factor 3 (composed of a cluster of short-chain dicarboxylacylcarnitine [SCDA] metabolites [similar to our previous studies [4, 5]]), were used as the quantitative traits for GWAS. Eigenstrat was used to define principal components (PCs) in GWAS. Four eigenvectors were used as PCs in whites, two in blacks, and seven in the “other” race category. Race-stratified linear regression models for each SNP (additive and dominant), adjusted for age, sex, race-specific PCs and metabolite batch, were constructed using PLINK [31]. Race-stratified results were also combined with meta-analysis using METAL [32]. Genomic inflation factors (λ) were <1.0. Significant SNPs were defined as those showing genomewide significance (p<10^−6^) in the discovery cohort and nominal association (p<0.05, unadjusted for multiple comparisons) in the validation cohort. Significant SNPs were then: (1) analyzed using meta-analysis of the cohorts using METAL [32]; (2) tested for association with metabolite factor levels after adjustment for glomerular filtration rate and in multivariable models (adjusted for BMI, hypertension, CAD, diabetes, left ventricular ejection fraction, dyslipidemia, smoking and renal disease); and (3) tested for association with time-to-death using Cox-proportional hazards modeling in the combined cohorts. Expression quantitative trait loci (eQTL) analyses of SNPs and SCDA levels were conducted using linear regression adjusted for age, race, sex and batch. Gene Set Enrichment Analysis (GSEA) [11], using the Preranked tool, was used on the resultant p-values for each SNP or SCDA covariate effect on expression levels to identify enriched KEGG pathways. GWAS analyses were corrected for multiple comparisons based on the above defined genomewide significance; other analyses were not adjusted for multiple comparisons and nominal unadjusted p-values are reported, with a p≤0.05 considered statistically significant.

### Whole genome methylation profiling

For the methylation studies, we analyzed blood samples from a discovery cohort composed of 11 individuals from the combined CATHGEN cohorts who had the highest SCDA factor levels and 12 individuals with the lowest levels; and a validation cohort of 12 individuals with the next highest SCDA factor levels and 11 individuals with the next lowest levels; all 46 individuals were selected from those with RNA expression microarray data also available. DNA was isolated from blood mononuclear cells and sodium bisulfite treated prior to being prepped for analysis on the Illumina HumanMethylation 450K BeadChip following the manufacturer’s guidelines, using the Zymo EZ DNA Methylation Kit using manufacture’s protocol (Zymo Research Corporation Irvine, California USA). The alternative incubation condition recommended if using the Illumina Infinium Methylation Assay was used (supplied in the manufacturer’s instruction manual appendix). Converted DNA was amplified, fragmented and hybridized to the Human Methylation27, RevB bead chip pool of allele-differentiating oligonucleotides.

We removed probes with detection p-value>.05 in >10% of samples, data based on fewer than three beads, and probes previously identified as cross-reactive with other genomic locations [33]. Samples were checked for gender mismatch using principal components analysis (PCA) of probes on chromosome X and assay controls were inspected to ensure good performance on all samples. After QC, the original group of 485K probes was reduced to 473K probes. Color bias correction and background adjustment were performed using *lumi* [34], followed by quantile normalization of methylated, unmethylated, type I and type II probes separately using *wateRmelon* [35]. Finally, we used Beta Mixture Quantile dilation (BMIQ) for intra-array normalization [36]. After preprocessing, overall methylation levels (β) were calculated as the ratio of methylated to total signal (i.e. β = M / (M + U)) where M is the methylated signal intensity for a probe, U is the unmethylated signal intensity, and β therefore ranges from 0 (unmethylated) to 1 (methylated). Δβ was calculated as the mean methylation difference between the high and low SCDA groups at each probe. To identify candidate regions of interest, we prioritized probes with |Δβ|>0.10 in the discovery set (N = 1287). After removing probes with a common SNP (MAF>.01) in the CpG or single-base extension site, we filtered to known genes containing at least two probes each with |Δβ|>0.10 within a 1 kB region (n = 97 probes in 28 genes). Finally, we restricted our probes with probes with |Δβ|>0.10 and the same direction of effect in both datasets (i.e. hypermethylation in high SCDA samples versus low, three genes). Although our primary criteria for follow-up were Δβ values and the presence of multiple correlated probes in a gene, we also tested for differential methylation using linear models and empirical Bayes methods as implemented in *limma* [37]. Our standard model adjusted for estimates of cell-type proportions present in each sample using the method of Houseman, et al. [38]; we also ran a sensitivity analysis that additionally included age, sex and race.

### Biochemical characterization of C6-DC peak

Adipoyl carnitine and 3-methylglutaryl carnitine were synthesized from carnitine chloride and the corresponding cyclic acid anhydride according to the method of Johnson [13]. Products were confirmed by mass spectrometry. The liquid chromatography (LC)-MS/MS method of Maeda et al. [12] was extensively modified. Acylcarnitines were derivatized to butyl esters. The analytical platform was converted to a UPLC format using an Acquity UPLC HSS T3 column and the ion pairing reagent was changed to triethyl ammonium acetate. The carnitines were eluted using a linear gradient using water as solvent A and 95/5 v/v acetonitrile/water as solvent B starting at 20% B.

## Supporting Information

S1 Fig Overall study design and study flow. (TIF)

S2 Fig Q-Q plots of genome-wide association results for metabolite factor 1 (MCA). Displayed are Q-Q plots for GWAS in the discovery cohort (adjusted for age, sex and PC-factors), (A) additive model, whites only; (B) dominant model, whites only; (C) additive model, blacks only; (D) dominant model, blacks only; (E) additive model, races combined; (F) dominant model, races combined.
(TIF)

S3 Fig Q-Q plots for genomewide association results for metabolite factor 2 (LCDA). Displayed are Q-Q plots for GWAS in the discovery cohort (adjusted for age, sex and PC-factors), (A) additive model, whites only; (B) dominant model, whites only; (C) additive model, blacks only; (D) dominant models, blacks only; (E) additive model, races combined; (F) dominant model, races combined.
(TIF)

S4 Fig Q-Q plots for genomewide association results for metabolite factor 3 (SCDA). Displayed are Q-Q plots for GWAS in the discovery cohort (adjusted for age, sex and PC-factors), (A) additive model, whites only; (B) dominant model, whites only; (C) additive model, blacks only; (D) dominant models, blacks only; (E) additive model, races combined; (F) dominant model, races combined.
(TIF)

S5 Fig Locus Zoom plots of SCDA (factor 3) mQTL. Displayed are LocusZoom plots with -log10(p-value) (left Y-axis) and LD (right Y-axis), discovery cohort: **(A)** *RSBN1L*, additive model, blacks only; **(B)** *FBXO25*|*ERICH1*, additive model, blacks only; **(C)** *FREM2|STOML3*, dominant model, race meta-analysis; **(D)** *SLC22A23|PXCD1*, dominant model, race meta-analysis.
(TIF)

S6 Fig Distribution of differentially methylated probes in individuals at extremes of SCDA levels. Displayed are plots of the distribution of methylated CpG probes in 10 individuals with extremely low SCDA levels and 9 individuals with high SCDA levels. The x-axis displays the degree of differential methylation and the Y-axis displays the count for the number of probes.
(TIF)

S7 Fig Biochemical refinement of C6-DC SCDA metabolite. Displayed are levels of two isomers of the C6-DC acylcarnitine metabolite: adipoyl and 3-methylglutaryl carnitine, in human plasma samples from 29 individuals with the highest C6-DC acylcarnitines from our previous studies, showing that the predominant isomer accounting for the high C6-DC levels is the 3-methylglutaryl carnitine.
(TIF)

S1 Table Principal components analysis (PCA) in combined CATHGEN cohorts. Displayed are the 14 factors identified through PCA in the combined discovery and validation CATHGEN cohorts (total N = 3512), with an annotated description of the top metabolites loaded for a given factor, and a list of the individual metabolites with the highest factors loads for each factor (absolute value of factor load >0.4).
(DOCX)

S2 Table Factor loads for individual metabolites in the SCDA PCA-derived factor. (DOCX)

S3 Table Extended phenotypic analyses of top genetic variants identified from SCDA GWAS. Presented are results for the association between our most significant SCDA GWAS genetic variants after adjustment for glomerular filtration rate (GFR); in a multivariable model adjusted for cardiovascular risk factors; and for time-to-event analyses for the relationship between genetic variants and incident cardiovascular events.
(DOCX)

S4 Table Differential expression analyses for top GWAS SNPs. This table displays p-values for the analysis of differential expression using an additive model for the top SNPs identified from the SCDA GWAS, for *cis*-acting transcripts, and for the individual *trans*-acting transcripts for each SNP.
(DOCX)
