# Supplementary figures and images for: Metabolomic Quantitative Trait Loci (mQTL) Mapping Implicates the Ubiquitin Proteasome System in Cardiovascular Disease Pathogenesis
Source: PLoS Genet. 2015 Nov 5;11(11):e1005553. doi: 10.1371/journal.pgen.1005553 (PMC4634848; doi:10.1371/journal.pgen.1005553)

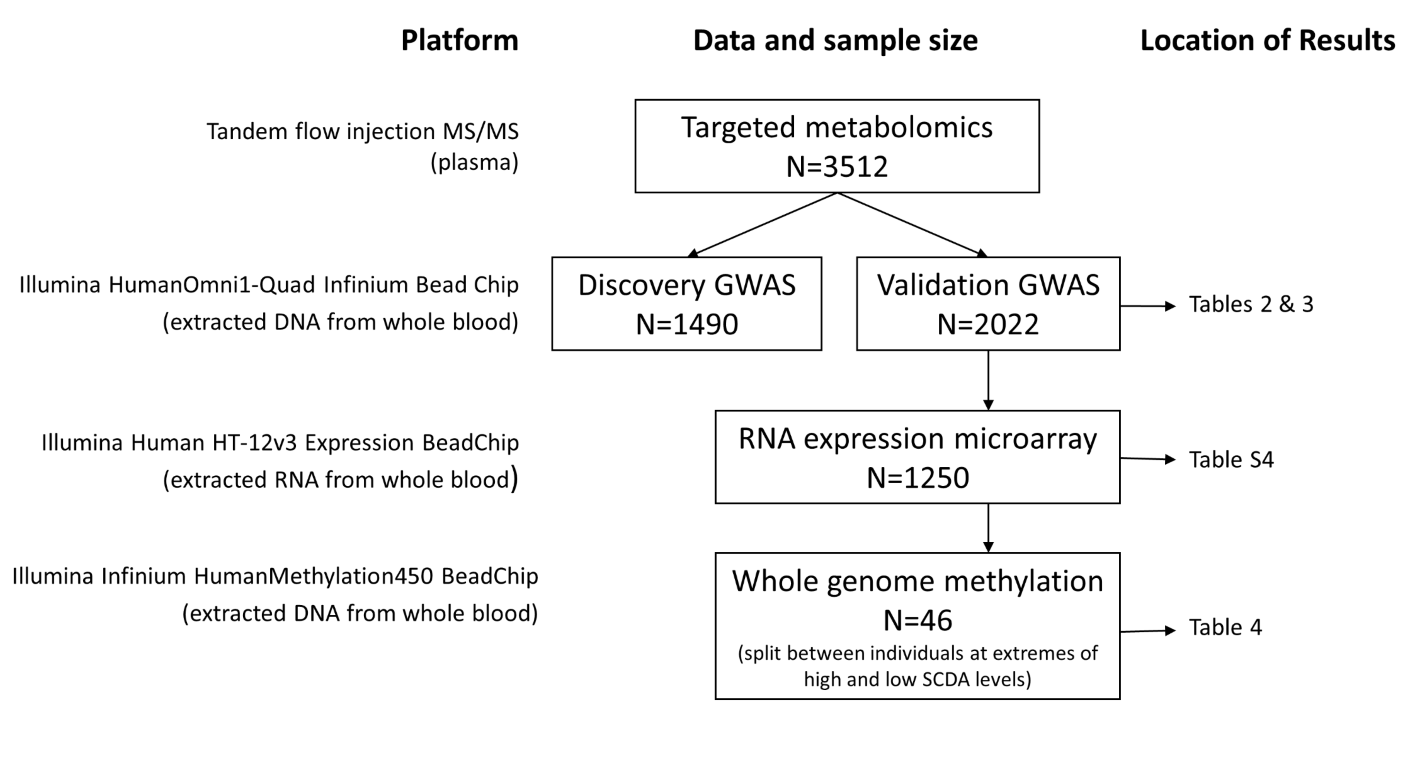

Supplement: S1 Fig — (TIF) [file pgen.1005553.s001.tif]

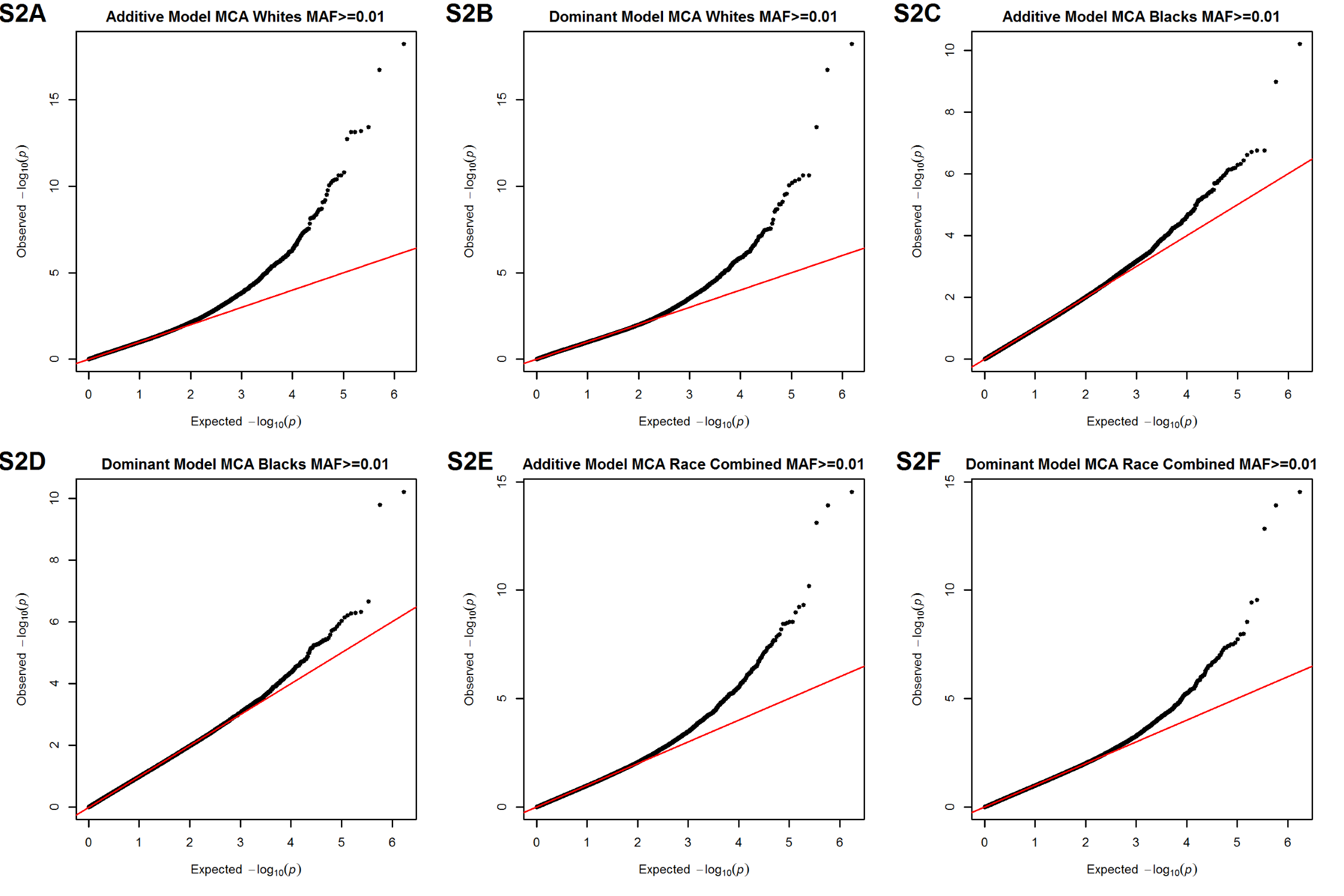

Supplement: S2 Fig — Displayed are Q-Q plots for GWAS in the discovery cohort (adjusted for age, sex and PC-factors), (A) additive model, whites only; (B) dominant model, whites only; (C) additive model, blacks only; (D) dominant model, blacks only; (E) additive model, races combined; (F) dominant model, races combined. (TIF) [file pgen.1005553.s002.tif]

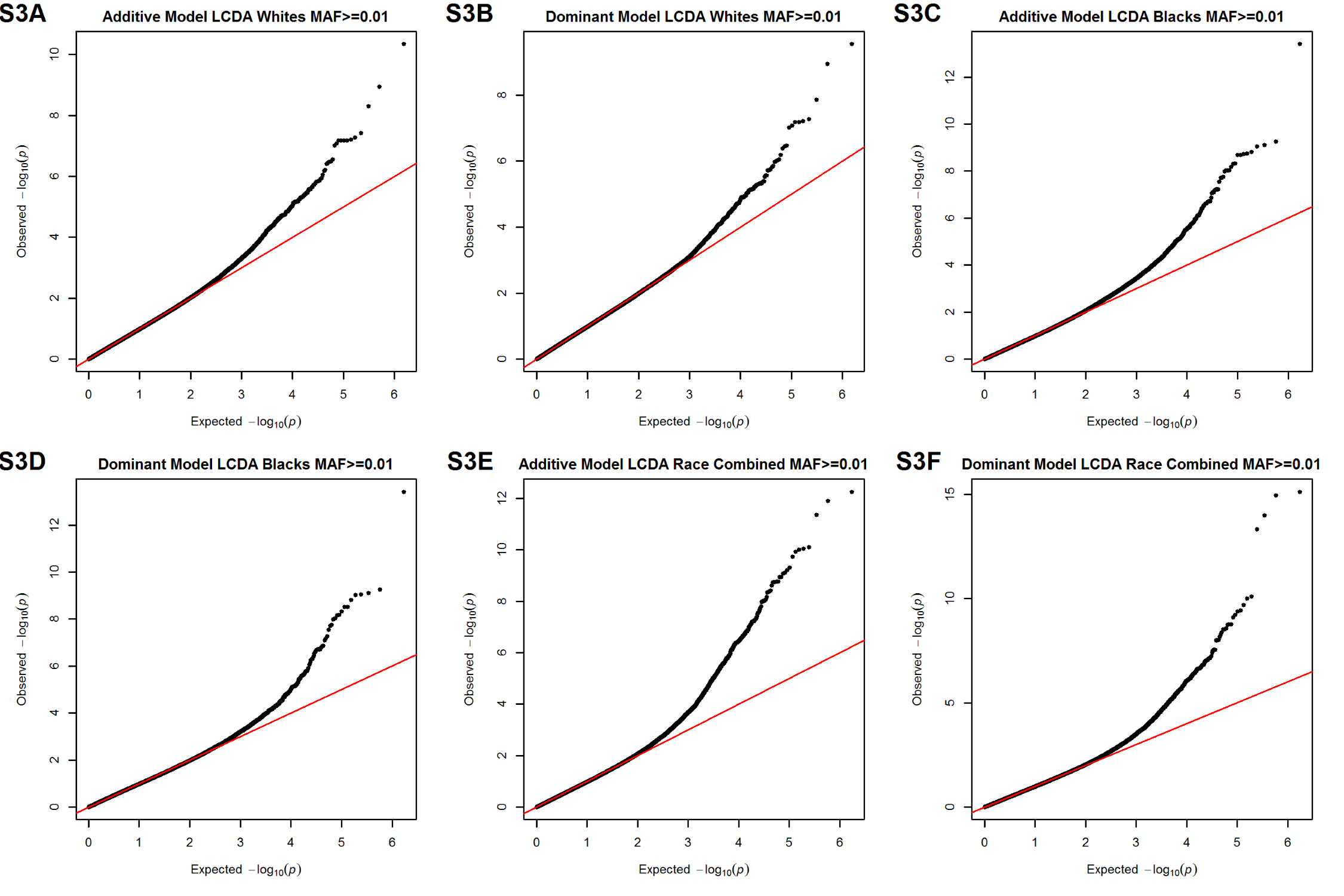

Supplement: S3 Fig — Displayed are Q-Q plots for GWAS in the discovery cohort (adjusted for age, sex and PC-factors), (A) additive model, whites only; (B) dominant model, whites only; (C) additive model, blacks only; (D) dominant models, blacks only; (E) additive model, races combined; (F) dominant model, races combined. (TIF) [file pgen.1005553.s003.tif]

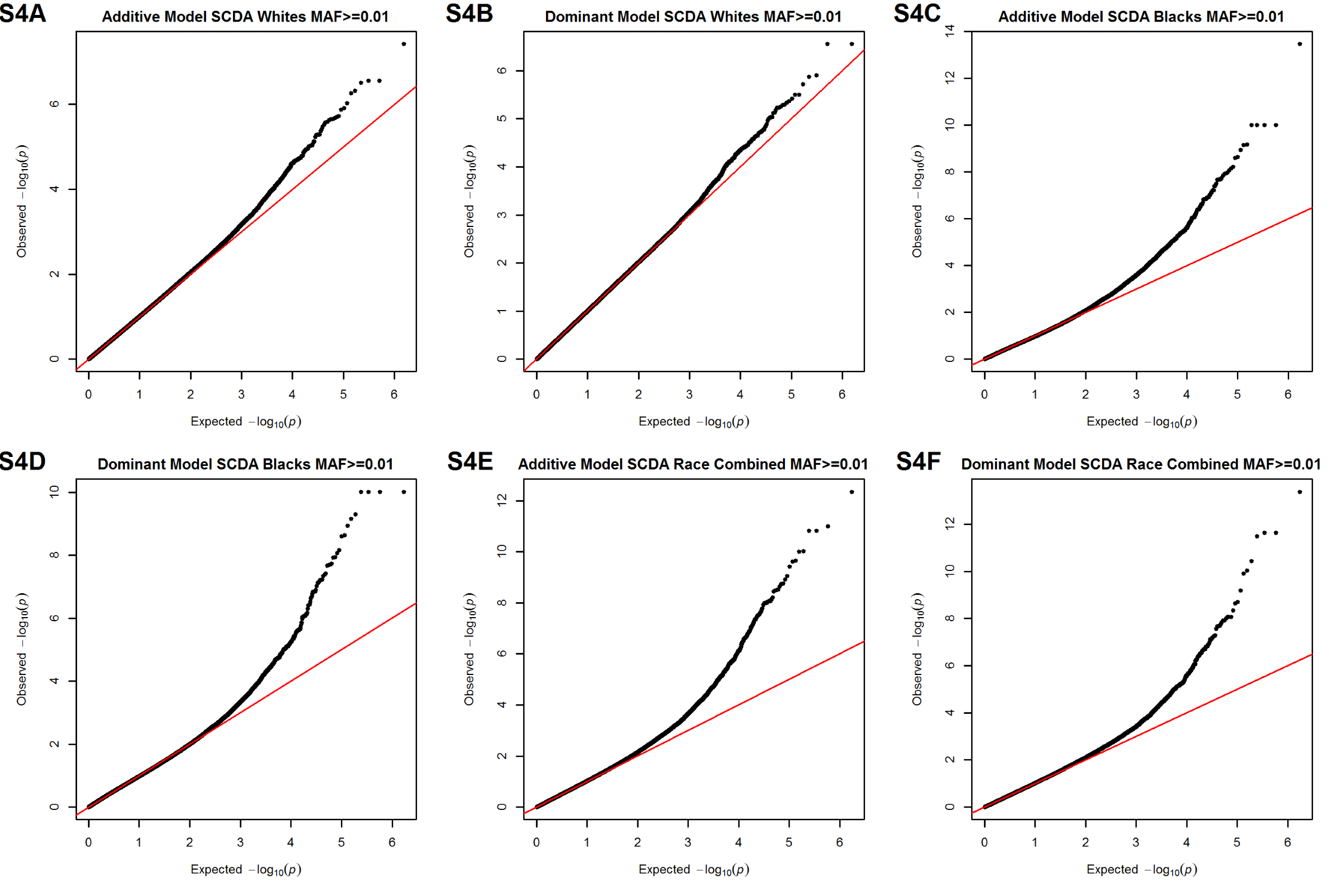

Supplement: S4 Fig — Displayed are Q-Q plots for GWAS in the discovery cohort (adjusted for age, sex and PC-factors), (A) additive model, whites only; (B) dominant model, whites only; (C) additive model, blacks only; (D) dominant models, blacks only; (E) additive model, races combined; (F) dominant model, races combined. (TIF) [file pgen.1005553.s004.tif]

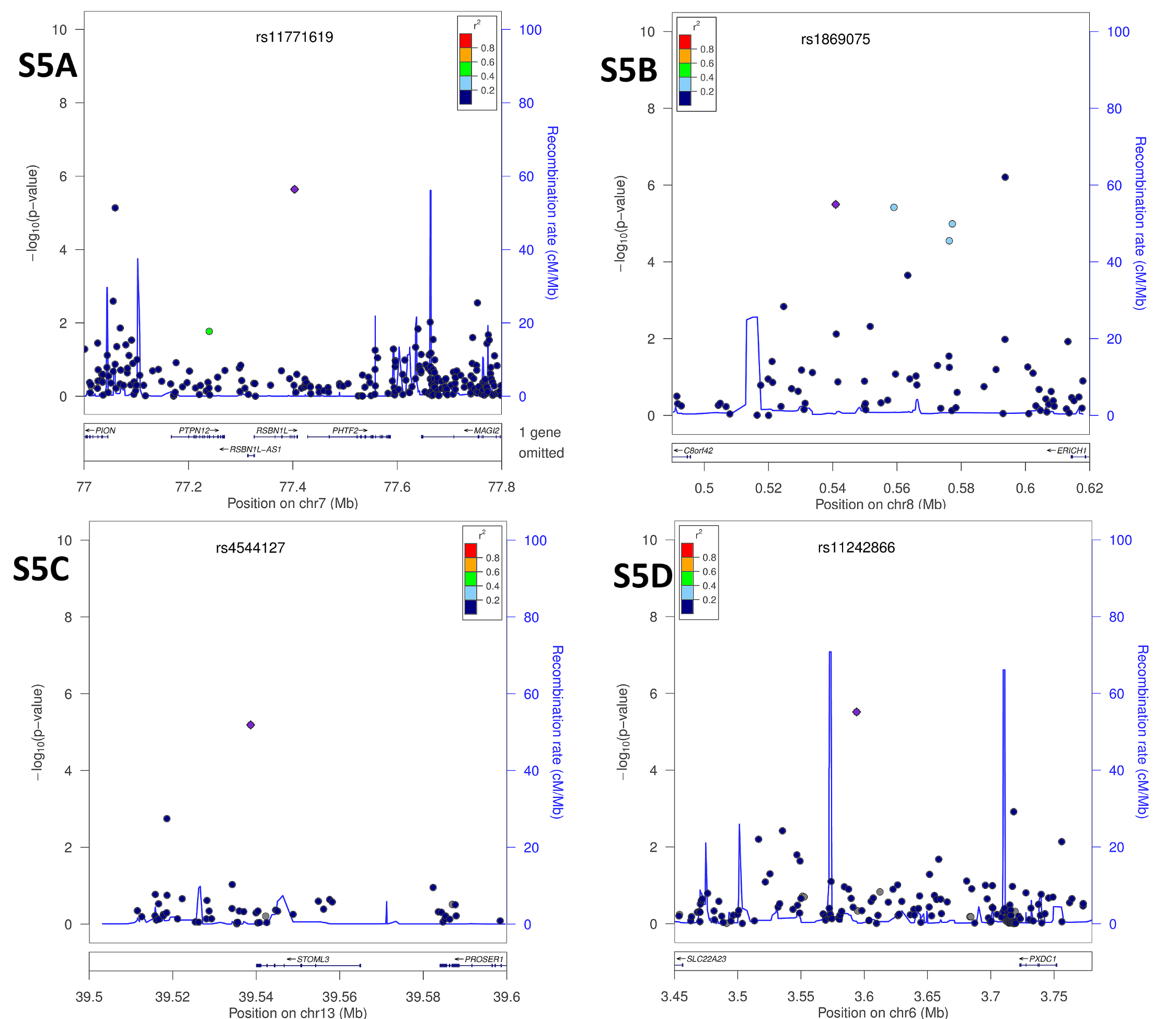

Supplement: S5 Fig — Displayed are LocusZoom plots with -log10(p-value) (left Y-axis) and LD (right Y-axis), discovery cohort: (A) RSBN1L, additive model, blacks only; (B) FBXO25|ERICH1, additive model, blacks only; (C) FREM2|STOML3, dominant model, race meta-analysis; (D) SLC22A23|PXCD1, dominant model, race meta-analysis. (TIF) [file pgen.1005553.s005.tif]

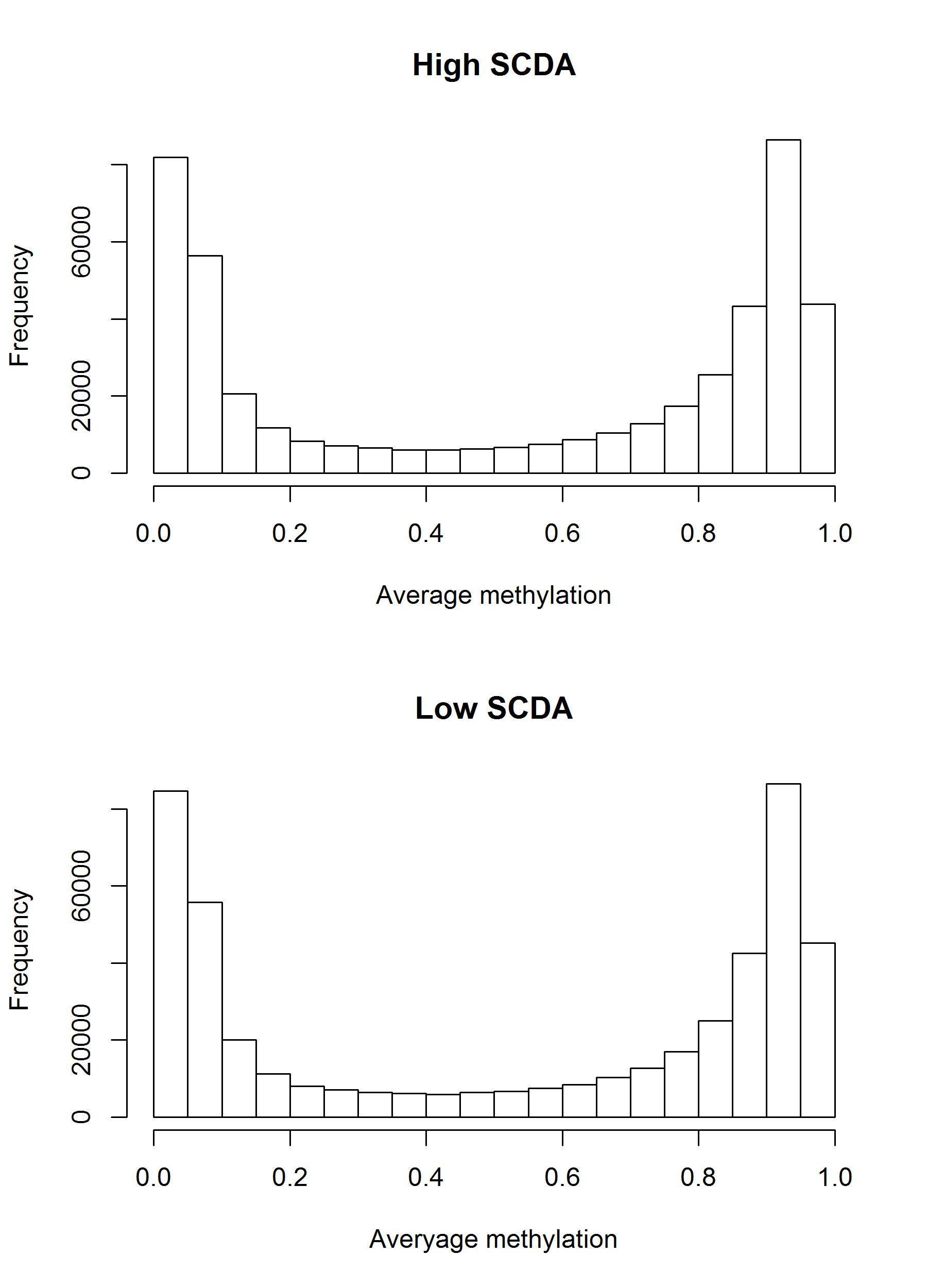

Supplement: S6 Fig — Displayed are plots of the distribution of methylated CpG probes in 10 individuals with extremely low SCDA levels and 9 individuals with high SCDA levels. The x-axis displays the degree of differential methylation and the Y-axis displays the count for the number of probes. (TIF) [file pgen.1005553.s006.tif]

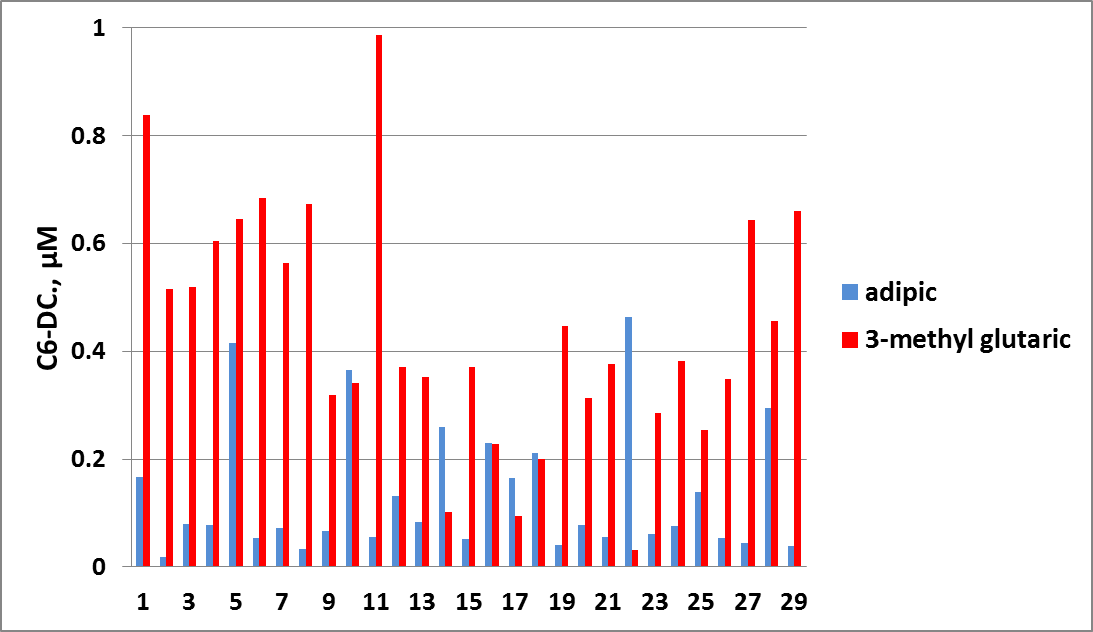

Supplement: S7 Fig — Displayed are levels of two isomers of the C6-DC acylcarnitine metabolite: adipoyl and 3-methylglutaryl carnitine, in human plasma samples from 29 individuals with the highest C6-DC acylcarnitines from our previous studies, showing that the predominant isomer accounting for the high C6-DC levels is the 3-methylglutaryl carnitine. (TIF) [file pgen.1005553.s007.tif]
