# Supplementary material for: Metabolomic Quantitative Trait Loci (mQTL) Mapping Implicates the Ubiquitin Proteasome System in Cardiovascular Disease Pathogenesis
Source: PLoS Genet. 2015 Nov 5;11(11):e1005553. doi: 10.1371/journal.pgen.1005553 (PMC4634848; doi:10.1371/journal.pgen.1005553)
Supplement: S1 Table — Displayed are the 14 factors identified through PCA in the combined discovery and validation CATHGEN cohorts (total N = 3512), with an annotated description of the top metabolites loaded for a given factor, and a list of the individual metabolites with the highest factors loads for each factor (absolute value of factor load >0.4). (DOCX) [file pgen.1005553.s008.docx]

**Table S1. Principal components analysis (PCA) in combined CATHGEN cohorts.** Displayed are the 14 factors identified through PCA in the combined discovery and validation CATHGEN cohorts (total N=3512), with an annotated description of the top metabolites loaded for a given factor, and a list of the individual metabolites with the highest factors loads for each factor (absolute value of factor load >0.4).

| **Factor** | **Description** | **Component Metabolites** |
| --- | --- | --- |
| **1** | Medium-Chain Acylcarnitines | C8, C10:1, C10, C12:1, C12, C14:2, C14:1, C14, C16:1 acylcarnitines |
| **2** | Long-Chain Dicarboxyl Acylcarnitines | C12-OH/C10-DC, C14-OH/C12-DC, C16-OH/C14-DC, C18:1-OH/C16:1-DC, C18-OH/C16-DC, C20, C20:1-OH/C18:1-DC, C20-OH/C18-DC acylcarnitines |
| **3** | Short-Chain Dicarboxyl Acylcarnitines | Citrulline, Ci4-DC/C4-DC, C5-DC, C6-DC, C10-OH/C8-DC, C12-OH/C10-DC, C6:1-DC/C8:1-OH, C8:1-DC acylcarnitines |
| **4** | Long-Chain Acylcarnitines | C16, C18:2, C18:1, C18, C16:1-OH/C14:1-DC, C20:4 acylcarnitines |
| **5** | Ketone Related | Alanine, C2 and C4-OH acylcarnitines, β-hydroxybutyrate, ketones |
| **6** | Medium-Chain Acylcarnitines | C8:1, C10:3, C10:2, C10:1 acylcarnitines |
| **7** | Branched-Chain AA | Valine, leucine/isoleucine, methionine, phenylalanine, tyrosine |
| **8** | Urea Cycle Amino Acids | Glycine, serine, proline, methionine, ornithine, arginine, C5:1 acylcarnitine |
| **9** | C3 – C5’s | C3, C4/Ci4, C5 acylcarnitines |
| **10** | Miscellaneous | Aspartate/aspartic acid, C5-OH/C3-DC acylcarnitine |
| **11** | Miscellaneous | Histidine, arginine, C5:1, C18:2-OH acylcarnitines |
| **12** | Miscellaneous | Valine, glutamic acid/glutamine |
| **13** | NEFA | Alanine, profile, nonesterified fatty acids |
| **14** | Miscellaneous | C22 acylcarnitine |
