# Supplementary material for: Metabolomic Quantitative Trait Loci (mQTL) Mapping Implicates the Ubiquitin Proteasome System in Cardiovascular Disease Pathogenesis
Source: PLoS Genet. 2015 Nov 5;11(11):e1005553. doi: 10.1371/journal.pgen.1005553 (PMC4634848; doi:10.1371/journal.pgen.1005553)
Supplement: S2 Table — (DOCX) [file pgen.1005553.s009.docx]

**Table S2. Factor loads for individual metabolites in the SCDA PCA-derived factor (factor 3).**

| Metabolite Symbol | Trivial name(s) | Factor 3 Load |
| --- | --- | --- |
| Gly | Glycine | -0.020542 |
| Ala | Alanine | -0.045373 |
| Ser | Serine | -0.030355 |
| Pro | Proline | 0.012627 |
| Val | Valine | -0.017171 |
| Leu/Ile | Leucine or Isoleucine | 0.028203 |
| Met | Methionine | -0.009760 |
| His | Histidine | 0.021233 |
| Phe | Phenylalanine | 0.059089 |
| Tyr | Tyrosine | 0.020944 |
| Asx | Asparagine or Aspartic acid | 0.026357 |
| Glx | Glutamine or Glutamic acid | 0.010410 |
| Ornithine | Ornithine | -0.002496 |
| Cit | Citrulline | 0.094248 |
| Arg | Arginine | 0.002358 |
| C2 | Acetyl carnitine | -0.065536 |
| C3 | Propionyl carnitine | -0.062477 |
| C4/Ci4 | Butyryl carnitine or Isobutyryl carnitine | -0.012459 |
| C5:1 | Tiglyl carnitine | 0.013343 |
| C5's | Isovaleryl carnitine, 3-methylbutyryl carnitine or 2-Methylbutyryl carnitine | 0.026780 |
| C4-OH | 3-Hydroxy-butyryl carnitine, β-hydroxy butyryl carnitine | -0.035205 |
| C5-OH/C3-DC | 3-Hydroxy-isovaleryl carnitine or Malonyl carnitine | -0.012725 |
| Ci4-DC/C4-DC | Methylmalonyl carnitine or Succinyl carnitine | 0.165934 |
| C8:1 | Octenoyl carnitine | -0.084161 |
| C8 | Octanoyl carnitine | -0.050216 |
| C5-DC | Glutaryl carnitine | 0.284739 |
| C6-DC/C8-OH | Adipoyl carnitine | 0.261498 |
| C10:3 | Decatrienoyl carnitine | -0.052679 |
| C10:2 | Decadienoyl carnitine | -0.036469 |
| C10:1 | Decenoyl carnitine | -0.078049 |
| C10 | Decanoyl carnitine | -0.014241 |
| C10-OH/C8-DC | 3-Hydroxy-decanoyl carnitine or Suberoyl carnitine | 0.115976 |
| C12:1 | Dodecenoyl carnitine | -0.040441 |
| C12 | Lauroyl carnitine | 0.035775 |
| C12-OH/C10-DC | 3-Hydroxy-dodecanoyl carnitine or Sebacoyl carnitine | 0.102662 |
| C14:2 | Tetradecadienoyl carnitine | -0.025325 |
| C14:1 | Tetradecenoyl carnitine | -0.015596 |
| C14 | Myristoyl carnitine | 0.011565 |
| C14:1-OH | 3-Hydroxy-tetradecenoyl carnitine | 0.029215 |
| C14-OH/C12-DC | 3-Hydroxy-tetradecanoyl carnitine or Dodecanedioyl carnitine | 0.001023 |
| C16 | Palmitoyl carnitine | 0.018086 |
| C16-OH/C14-DC | 3-Hydroxy-hexadecanoyl carnitine or Tetradecanedioyl carnitine | -0.027087 |
| C18:2 | Linoleyl carnitine | -0.002976 |
| C18:1 | Oleyl carnitine | 0.032270 |
| C18 | Stearoyl carnitine | 0.020729 |
| C18:1-OH | 3-Hydroxy-octadecenoyl carnitine | -0.025705 |
| C18-OH/C16-DC | 3-Hydroxy-octadecanoyl carnitine or Hexadecanedioyl carnitine, thapsoyl carnitine | -0.030858 |
| C20 | Arachidoyl carnitine, eicosanoyl carnitine | -0.016203 |
| C18:1-DC | Octadecenedioyl carnitine | -0.021031 |
| C20-OH/C18-DC | 3-Hydroxy-eicosanoyl carnitine or Octadecanedioyl carnitine | -0.041326 |
| C22 | Behenoyl carnitine, docosanoyl carnitine | -0.008636 |
| C8:1-OH/C6:1-DC | 3-Hydroxy- octenoyl carnitine or Hexenedioyl carnitine | 0.192622 |
| C10:1-OH/C8:1-DC | 3-Hydroxy-decenoyl carnitine/Octenedioyl carnitine | 0.198564 |
| C16:2 | Hexadecadienoyl carnitine | -0.039812 |
| C16:1 | Palmitoleoyl carnitine | -0.018469 |
| C16:1-OH/C14:1-DC | 3-Hydroxy-palmitoleoyl carnitine or cis-5-Tetradecenedioyl carnitine | 0.007796 |
| C18:2-OH | 3-Hydroxy-linoleyl carnitine | 0.025373 |
| C20:4 | Arachidonoyl carnitine | -0.001175 |
| HBUT | β-Hydroxybutyrate | 0.025266 |
| KET | Total Ketones | 0.026695 |
| NEFA | Non-Esterified Fatty Acids | -0.003720 |
