# Supplementary material for: Metabolomic Quantitative Trait Loci (mQTL) Mapping Implicates the Ubiquitin Proteasome System in Cardiovascular Disease Pathogenesis
Source: PLoS Genet. 2015 Nov 5;11(11):e1005553. doi: 10.1371/journal.pgen.1005553 (PMC4634848; doi:10.1371/journal.pgen.1005553)
Supplement: S3 Table — Presented are results for the association between our most significant SCDA GWAS genetic variants after adjustment for glomerular filtration rate (GFR); in a multivariable model adjusted for cardiovascular risk factors; and for time-to-event analyses for the relationship between genetic variants and incident cardiovascular events. (DOCX) [file pgen.1005553.s010.docx]

**Table S3: Extended phenotypic analyses of top genetic variants identified from SCDA GWAS.** Presented are results for the association between our most significant SCDA GWAS genetic variants after adjustment for glomerular filtration rate (GFR); in a multivariable model adjusted for cardiovascular risk factors; and for time-to-event analyses for the relationship between genetic variants and incident cardiovascular events.

| **Gene** | **SNP** | **Race** | **GFR* Adjusted Model** | **Multivariable p-value†** | **Time to Event p**** | **Time to Event Adjusted SCDA p***** | **Event rate by genotype^††^** |
| --- | --- | --- | --- | --- | --- | --- | --- |
| *OLFM4 \| SUGT1* | rs17573278 | Whites | 2.7x10^-6^ | 3.0x10^-6^ | NS |  | 34%, 32%, 33% |
| *OLFM4 \| SUGT1* | rs9591507 | Whites | 2.4x10^-6^ | 2.9x10^-6^ | NS | NS | 34%, 32%, 33% |
| *OLFM4 \| SUGT1* | rs9591507 | Races combined | 2.6x10^-7^ | 3.4x10^-6^ | NS | NS | 33%, 36%, 24% |

| *COL23A1* | rs17081346 | Whites | 4.9x10^-6^ | 7.3x10^-7^ | NS | NS | 34%, 28%, N/A |
| --- | --- | --- | --- | --- | --- | --- | --- |
| *COL23A1* | rs17052428 | Whites | 4.9x10^-6^ | 1.4x10^-6^ | NS | NS | 34%, 27%, N/A |
| *PLA2G4A \| FAM5C* | rs16829453 | Whites | 7.1x10^-5^ | 5.3x10^-6^ | NS | NS | 33%, 40%, 0% |
| *OLFM4 \| SUGT1* | rs9285184 | Whites | 4.1x10^-6^ | 3.9x10^-6^ | NS | NS | 34%, 32%, 33% |
| *OLFM4 \| SUGT1* | rs894840 | Whites | 5.0x10^-5^ | 6.9x10^-5^ | NS | NS | 34%, 34%, 38% |

| *HERC1* | rs2228513 | Whites | 2.0x10^-10^ | 3.8x10^-7^ | 0.04 | 0.17 | 33%, 36%, 83% |
| --- | --- | --- | --- | --- | --- | --- | --- |
| *HERC1* | rs2228513 | Races combined | 5.6x10^-07^ | 2.9x10^-4^ | 0.05 | 0.15 | 33%, 35%, 83% |
| *USP3* | rs10450989 | Whites | 6.6x10^-11^ | 1.8x10^-7^ | 0.05 | 0.19 | 34%, 35%, 83% |
| *USP3* | rs10450989 | Races Combined | 2.7x10^-7^ | 1.9x10^-4^ | 0.06 | 0.17 | 33%, 35%, 83% |
|  |  |  |  |  |  |  |  |
| *RSBN1L* | rs11771619 | Blacks | 6.3x10^-6^ | 2.1x10^-6^ | NS | NS | 33%, 36%, 100% |
| *FBXO25* \| *ERICH1* | rs1869075 | Blacks | 2.0x10^-4^ | 0.08 | 2.5x10^-4^ | 2.7x10^-3^ | 31%, 45%, 50% |
| *FBXO25* \| *ERICH1* | rs1869075 | Races Combined | 0.07 | 0.13 | 0.31 | 0.44 | 33%, 35%, 33% |
|  |  |  |  |  |  |  |  |
| *ELF3 \| GPR37L1* | rs12139192 | Blacks | 1.6x10^-4^ | 4.0x10^-4^ | 0.06 | NS | 33%, 38%, 33% |
|  |  |  |  |  | NS |  |  |
| *RRM1 \| STIM1* | rs930491 | Races combined | 1.8x10^-3^ | 0.01 | NS | NS | 33%, 36%, 50% |
| *RRM1 \| STIM1* | rs11827377 | Races combined | 2.0x10^-3^ | 0.01 | NS | NS | 33%, 37%, 50% |
| *STON2 \| SEL1L* | rs12589750 | Races combined | 4.7x10^-3^ | 0.02 | NS | 0.10 | 34%, 28%, 20% |
| *STON2 \| SEL1L* | rs3853422 | Races combined | 2.7x10^-3^ | 0.06 | NS | NS | 34%, 29%, 33% |
|  |  |  |  |  |  |  |  |

| *SULF2\|PREX1* | rs1886848 | Races combined | 2.9x10^-7^ | 2.6x10^-8^ | NS | NS | 34%, 35%, 50% |
| --- | --- | --- | --- | --- | --- | --- | --- |
| *RRM1 \| STIM1* | rs11826962 | Races combined | 5.3x10^-4^ | 0.01 | 0.03 | 0.07 | 33%, 38%, 100% |
| *SLC22A23\|PXDC1* | rs11242866 | Races combined | 6.3x10^-3^ | 0.01 | NS | NS | 33%, 40%, 50% |
| *CADM2 \| VGLL3* | rs6796873 | Races combined | 3.0x10^-3^ | 9.0x10^-3^ | 0.09 | NS | 34%, 30%, 44% |
| *C14orf105* | rs10139566 | Races combined | 5.0x10^-3^ | 0.04 | NS | NS | 33%, 37%, 41% |
| *RAMP1* | rs3769047 | Races combined | 0.13 | 0.4 | NS | NS | 34%, 32%, 50% |

| *FREM2 \| STOML3* | rs4544127 | Races combined | 0.15 | 0.2 | 0.06 | 0.11 | 33%, 42%, 50% |
| --- | --- | --- | --- | --- | --- | --- | --- |

*Adjusted for age, sex, PC factors and glomerular filtration rate (GFR). GFR as calculated using Cockroft-Gault formula, discovery and validation cohorts meta-analysis, additive model.
†Discovery and validation cohorts combined with meta-analysis, additive model, adjusted for age, sex, PC factors, BMI, hypertension, coronary artery disease, diabetes, ejection fraction, dyslipidemia, sex, smoking, renal disease.
**P-value for Cox proportional hazards modeling for genotype by time-to-death, discovery and validation cohorts combined, adjusted for age, sex, and PC factors.

***P-value for Cox proportional hazards modeling for genotype by time-to-death, discovery and validation cohorts combined, adjusted for SCDA factor levels, age, sex, and PC factors.
††Event rate (death or MI) stratified by genotype (0, 1, 2 minor allele carriers), for racial category specified.
NS: not significant
