# Supplementary material for: Metabolomic Quantitative Trait Loci (mQTL) Mapping Implicates the Ubiquitin Proteasome System in Cardiovascular Disease Pathogenesis
Source: PLoS Genet. 2015 Nov 5;11(11):e1005553. doi: 10.1371/journal.pgen.1005553 (PMC4634848; doi:10.1371/journal.pgen.1005553)
Supplement: S4 Table — This table displays p-values for the analysis of differential expression using an additive model for the top SNPs identified from the SCDA GWAS, for cis-acting transcripts, and for the individual trans-acting transcripts for each SNP. (DOCX) [file pgen.1005553.s011.docx]

**Table S4. Differential expression analyses for top GWAS SNPs.** This table displays p-values for the analysis of differential expression using an additive model for the top SNPs identified from the SCDA GWAS, for *cis*-acting transcripts, and for the individual *trans*-acting transcripts for each SNP.

| **Gene** | **SNP** | ***Cis* transcript p-value*** | **Top 10 differentially expressed individual *trans*-acting transcripts (p-values)** |
| --- | --- | --- | --- |
| *OLFM4 \| SUGT1* | rs17573278 | 0.01 | 3.82132E-06 MFSD11 (major facilitator superfamily domain containing 11)  3.9048E-05 ENSA (endosulfine alpha)  6.0435E-05 HS.549989  6.20088E-05 HS.371006  6.96085E-05 UTP23 (small subunit (SSU) processome component)  0.000120966 CXCR5 (chemokine (C-X-C motif) receptor 5)  0.00016497 LOC728518  0.000181285 C17ORF90  0.000187735 SSH1 (slingshot protein phosphatase 1)  0.000219899 ANXA11 (annexin A11) |
| *OLFM4 \| SUGT1* | rs9591507 | 0.006 | 1.28807E-05 UTP23 (small subunit (SSU) processome component)  3.37396E-05 SSH1 (slingshot protein phosphatase 1)  0.00017543 CTNNB1 (catenin (cadherin-associated protein), beta 1, 88kDa)  0.000348076 FBXW7 (F-box and WD repeat domain containing 7, E3 ubiquitin protein ligase)  0.000377061 LOC286310  0.000493913 MFSD11 (major facilitator superfamily domain containing 11)  0.000528721 ZNF699  0.000648408 PTPRC (protein tyrosine phosphatase, receptor type, C)  0.000742666 LOC644144  0.000840972 HS.97408 |
| *OLFM4 \| SUGT1* | rs894840 | 0.01 | 9.943E-06 RN7SK (RNA; 7SK small nuclear)  2.9363E-05 B3GALT4 (UDP-Gal:betaGlcNAc beta 1;3-galactosyltransferase; polypeptide 4)  8.7818E-05 CDC42SE1 (CDC42 small effector)  0.00018964 RABAC1 (Rab acceptor 1 (prenylated))  0.00035175 TTC1 (tetratricopeptide repeat domain 1)  0.00044748 KRT72 (keratin 72)  0.00047086 FSCN1 (fascin homolog 1; actin-bundling protein (Strongylocentrotus purpuratus))  0.0007322 PTAFR (platelet-activating factor receptor)  0.00101701 ACSL5 (acyl-CoA synthetase long-chain family member 5)  0.00105124 UTP23 (small subunit (SSU) processome component; homolog (yeast)) |
| *OLFM4 \| SUGT1* | rs9285184 | 0.005 | 3.1219E-06 UTP23 (small subunit (SSU) processome component; homolog (yeast))  6.5956E-06 SSH1 (slingshot homolog 1 (Drosophila))  2.4088E-05 ANXA11 (annexin A11)  4.0997E-05 FLJ42957  0.00015937 FBXW7 (F-box and WD repeat domain containing 7)  0.00016244 XRN1 (5'-3' exoribonuclease 1)  0.00018855 B3GALT4 (UDP-Gal:betaGlcNAc beta 1;3-galactosyltransferase; polypeptide 4)  0.00020077 MFSD11 (major facilitator superfamily domain containing 11)  0.00020107 CXCR5 (chemokine (C-X-C motif) receptor 5)  0.00022388 ADAM17 (ADAM metallopeptidase domain 17 (tumor necrosis factor; alpha; converting enzyme)) |
| *HERC1* | rs2228513 | 0.16 | 0.000219305 USP39 (ubiquitin specific peptidase 39)  0.000255892 KIAA0664  0.000276197 ITPRIPL1 (inositol 1,4,5-trisphosphate receptor interacting protein-like 1)  0.000515724 PGGT1B (protein geranylgeranyltransferase type I, beta subunit)  0.00081543 NPCDR1 (nasopharyngeal carcinoma, down-regulated 1)  0.000941459 WDR22 (DCAF5 DDB1 and CUL4 associated factor 5)  0.001284715 LOC728554  0.001481333 RPS26L1 (ribosomal protein S26 pseudogene 11)  0.001508455 CYLD (cylindromatosis (turban tumor syndrome)  0.001604546 ZBTB7B (zinc finger and BTB domain containing 7B) |
| *USP3* | rs10450989 | 0.51 | 0.000415346 ITPRIPL1 (inositol 1,4,5-trisphosphate receptor interacting protein-like 1)  0.000421749 USP39 (ubiquitin specific peptidase 39)  0.000451914 KIAA0664  0.000546324 NPCDR1 (nasopharyngeal carcinoma, down-regulated 1)  0.000713335 CYLD (cylindromatosis (turban tumor syndrome))  0.000823877 MRPS30 (mitochondrial ribosomal protein S30)  0.001059736 PGGT1B (protein geranylgeranyltransferase type I, beta subunit)  0.001278082 LOC642161  0.001309435 LYSMD2 (LysM, putative peptidoglycan-binding, domain containing 2)  0.001404232 WDR22 (DCAF5 DDB1 and CUL4 associated factor 5) |
| *RSBN1L* | rs11771619 | 0.01 | 5.69578E-05 DUSP14 (dual specificity phosphatase 14)  8.7483E-05 SUSD5 (sushi domain containing 5)  0.000120731 BRP44L (brain protein 44-like)  0.00017439 CDAN1 (codanin 1)  0.000202716 OR4B1 (olfactory receptor, family 4, subfamily B, member 1)  0.000257383 RNASE4 (ribonuclease, RNase A family, 4)  0.000348536 HS.566890  0.000395503 SNRPC (small nuclear ribonucleoprotein polypeptide C)  0.000398385 LOC653253  0.00050466 ZNF511 (zinc finger protein 511) |
| *FBXO25* \| *ERICH1* | rs1869075 | 9.8x10^-5^ | 9.7868E-05 ERICH1 (glutamate-rich 1)  0.001776672 S100PBP (S100P binding protein)  0.003607347 GPR65 (G protein-coupled receptor 65)  0.00377641 NSUN4 (NOP2/Sun domain family, member 4)  0.005351906 TOR3A (torsin family 3, member A)  0.005530848 TAPBP (TAP binding protein (tapasin))  0.005574405 EIF1AX (eukaryotic translation initiation factor 1A, X-linked)  0.006079327 C7ORF28A  0.006166286 P76 (mannose-6-phosphate protein p76)  0.006217998 SHOC2 (soc-2 suppressor of clear homolog (C. elegans)) |
| *ELF3 \| GPR37L1* | rs12139192 | NA | 0.001210789 APOBEC3G (apolipoprotein B mRNA editing enzyme, catalytic polypeptide-like 3G)  0.001383219 SH3BGRL2 (SH3 domain binding glutamic acid-rich protein like 2)  0.002363442 MGC13005  0.002579649 TMEM51 (transmembrane protein 51)  0.002719163 CTTN (cortactin)  0.002883921 PGCP (plasma glutamate carboxypeptidase)  0.002939705 C10ORF76  0.003261817 APOBEC3F (apolipoprotein B mRNA editing enzyme, catalytic polypeptide-like 3F)  0.003332474 MGAM (maltase-glucoamylase (alpha-glucosidase))  0.004077693 PDIA6 (protein disulfide isomerase family A, member 6) |
| *RRM1 \|STIM1* | rs930491 | 0.56 | 2.0962E-05 VAMP3 (vesicle-associated membrane protein 3)  3.9473E-05 GRK6 (G protein-coupled receptor kinase 6)  4.82767E-05 INPP5A (inositol polyphosphate-5-phosphatase, 40kDa)  9.07639E-05 IGBP1 (immunoglobulin (CD79A) binding protein 1)  0.000248604 CDC42 (cell division cycle 42)  0.000260074 DNAJC7 (DnaJ (Hsp40) homolog, subfamily C, member 7)  0.000424009 RRAGD (Ras-related GTP binding D)  0.00047219 GTF3C6 (general transcription factor IIIC, polypeptide 6, alpha 35kDa)  0.000696829 UBTD1 (ubiquitin domain containing 1)  0.00071473 FAM46A (family with sequence similarity 46, member A) |
| *RRM1 \| STIM1* | rs11827377 | 0.56 | 2.0962E-05 VAMP3 (vesicle-associated membrane protein 3)  3.9473E-05 GRK6 (G protein-coupled receptor kinase 6)  4.82767E-05 INPP5A (inositol polyphosphate-5-phosphatase, 40kDa)  9.07639E-05 IGBP1 (immunoglobulin (CD79A) binding protein 1)  0.000248604 CDC42 (cell division cycle 42)  0.000260074 DNAJC7 (DnaJ (Hsp40) homolog, subfamily C, member 7)  0.000424009 RRAGD (Ras-related GTP binding D)  0.00047219 GTF3C6 (general transcription factor IIIC, polypeptide 6, alpha 35kDa)  0.000696829 UBTD1 (ubiquitin domain containing 1)  0.00071473 FAM46A (family with sequence similarity 46, member A) |
| *RRM1 \| STIM1* | rs11826962 | 0.26 | 3.3658E-05 VAMP3 (vesicle-associated membrane protein 3 (cellubrevin))  0.00037944 FCGR3B (Fc fragment of IgG; low affinity IIIb; receptor (CD16b))  0.00056757 RPS27A (ribosomal protein S27a)  0.00076261 DHX36 (DEAH (Asp-Glu-Ala-His) box polypeptide 36 )  0.00082794 SC4MOL (sterol-C4-methyl oxidase-like)  0.00082978 LOC642035  0.00090724 ERO1L (ERO1-like (S. cerevisiae))  0.00095968 LOC90120  0.00099971 SLA (Src-like-adaptor)  0.00162754 C20ORF30 (chromosome 20 open reading frame 30) |
| *STON2* | rs12589750 | NA | 1.39026E-06 UNC119 (unc-119 homolog (C. elegans))  3.53896E-06 LILRA2 (leukocyte immunoglobulin-like receptor, subfamily A (with TM domain), member 2)  3.46382E-05 RNPEP (arginyl aminopeptidase (aminopeptidase B))  6.21909E-05 N4BP2 (NEDD4 binding protein 2)  6.6553E-05 TGM3 (transglutaminase 3)  7.17697E-05 LSM14A (SCD6 homolog A (S. cerevisiae))  7.71145E-05 PANX2 (pannexin 2)  8.0975E-05 TPT1 (tumor protein, translationally-controlled 1)  9.35729E-05 ZNF467  0.000113926 FOS (FBJ murine osteosarcoma viral oncogene homolog) |
| *STON2 \| SEL1L* | rs3853422 | NA | 5.94096E-06 LOC728417  2.22524E-05 FCGR3B (Fc fragment of IgG, low affinity IIIb, receptor (CD16b)  2.85558E-05 LOC728417  3.2218E-05 C1ORF38  3.23666E-05 CDC42SE1 (CDC42 small effector 1)  6.82251E-05 IL4R (interleukin 4 receptor)  0.000101371 LIG3 (ligase III, DNA, ATP-dependent)  0.000129151 LOC201175  0.00019122 TAX1BP1 (Tax1 (human T-cell leukemia virus type I) binding protein 1)  0.000232555 CXCR4 (chemokine (C-X-C motif) receptor 4) |
| *FREM2 \| STOML3* | rs4544127 | NA | 3.4857E-05 ISG15 (ISG15 ubiquitin-like modifier)  9.95481E-05 UBE2D3 (ubiquitin-conjugating enzyme E2D 3)  0.000104456 EXTL3 (exostosin-like glycosyltransferase 3)  0.000178228 SCO2 (SCO2 cytochrome c oxidase assembly protein)  0.000247285 ATP5S (ATP synthase, H+ transporting, mitochondrial Fo complex, subunit s (factor B))  0.000362736 PPP1CB (protein phosphatase 1, catalytic subunit, beta isozyme)  0.000505676 IRF7 (interferon regulatory factor 7)  0.000632875 CDC42EP4 (CDC42 effector protein (Rho GTPase binding) 4)  0.000634144 IRF7 (interferon regulatory factor 7)  0.000667875 USF1 (upstream transcription factor 1) |
| *SULF2 \| PREX1* | rs1886848 | 0.0014 | 1.8976E-06 ITFG1 (integrin alpha FG-GAP repeat containing 1)  2.9271E-06 TRAF3IP2 (TRAF3 interacting protein 2)  3.4995E-06 C14ORF93  5.0015E-06 ATG7 (ATG7 autophagy related 7 homolog (S. cerevisiae))  6.1112E-06 SF3B2 (splicing factor 3b; subunit 2; 145kDa)  9.0935E-06 AK1 (adenylate kinase 1)  9.4968E-06 CABC1 (chaperone; ABC1 activity of bc1 complex homolog (S. pombe))  9.6619E-06 KLHL22 (kelch-like 22 (Drosophila))  9.6951E-06 RGS14 (regulator of G-protein signaling 14)  1.2447E-05 HSD17B4 (hydroxysteroid (17-beta) dehydrogenase 4) |
| *C14orf105* | rs10139566 | NA | 1.1173E-05 HS.82028  2.2073E-05 POLM (polymerase (DNA directed); mu)  4.0725E-05 LOC649841  5.8402E-05 LOC642035  7.4698E-05 STAG3L1 (stromal antigen 3-like 1)  7.9426E-05 CCT6A (chaperonin containing TCP1; subunit 6A (zeta 1))  0.00010958 POLR3C (polymerase (RNA) III (DNA directed) polypeptide C (62kD))  0.00011414 ADD3 (adducin 3 (gamma))  0.00011834 GGA3 (golgi associated; gamma adaptin ear containing; ARF binding protein 3)  0.00012988 ATF7IP (activating transcription factor 7 interacting protein) |
| *SLC22A23 \| PXDC1* | rs11242866 | NA | 8.5712E-05 NLRP12 (NLR family; pyrin domain containing 12)  0.00013415 SLC2A8 (solute carrier family 2 (facilitated glucose transporter); member 8)  0.00024186 GPR68 (G protein-coupled receptor 68)  0.00029858 SF1 (splicing factor 1 (SF1)  0.00043629 TMEM103 (transmembrane protein 103)  0.00078365 DPM1 (dolichyl-phosphate mannosyltransferase polypeptide 1)  0.00090133 EXOSC4 (exosome component 4)  0.00166889 LY9 (lymphocyte antigen 9)  0.00172613 TXNDC5 (thioredoxin domain containing 5)  0.00187173 TMEM50A (transmembrane protein 50A) |
| *ELF3 \| GPR37L1* | rs12139192 | NA | 0.00128307 APOBEC3G (apolipoprotein B mRNA editing enzyme; catalytic polypeptide-like 3G)  0.00145911 SH3BGRL2 (SH3 domain binding glutamic acid-rich protein like 2)  0.00241997 MGC13005  0.00275098 TMEM51 (transmembrane protein 51)  0.00282519 CTTN (cortactin)  0.00301006 C10ORF76  0.00313196 PGCP (plasma glutamate carboxypeptidase)  0.00334261 APOBEC3F (apolipoprotein B mRNA editing enzyme; catalytic polypeptide-like 3F)  0.00351275 MGAM (maltase-glucoamylase (alpha-glucosidase))  0.00386302 PDIA6 (protein disulfide isomerase family A; member 6) |
| *PLA2G4A \| FAM5C* | rs16829453 | NA | 2.5766E-05 TMEM93 (transmembrane protein 93  0.0004182 TOMM22 (translocase of outer mitochondrial membrane 22 homolog (yeast))  0.00083064 KLHL7 (kelch-like 7 (Drosophila))  0.00105423 B3GNT8 (UDP-GlcNAc:betaGal beta-1;3-N-acetylglucosaminyltransferase 8)  0.0011483 CSGLCA-T (chondroitin sulfate glucuronyltransferase)  0.00128121 KIAA1429  0.00137032 ALKBH7 (alkB; alkylation repair homolog 7 (E. coli))  0.00139886 PPIH (peptidylprolyl isomerase H (cyclophilin H))  0.00145334 SIGLEC7 (sialic acid binding Ig-like lectin 7)  0.00155918 PSMD6 (proteasome (prosome; macropain) 26S subunit; non-ATPase; 6) |
| *COL23A1* | rs17052428 | NA | 1.0718E-05 HS.562660  2.2315E-05 TMEM97 (transmembrane protein 97)  7.7962E-05 SAPS3 (SAPS domain family; member 3)  0.0002639 PAPOLA (poly(A) polymerase alpha)  0.00082388 HBG1 (hemoglobin; gamma A)  0.00088504 HBG2 (hemoglobin; gamma G)  0.00103242 C20ORF55  0.00111321 MAGMAS (mitochondria-associated protein involved in granulocyte-macrophage colony-stimulating factor signal transduction)  0.00131622 DVL2 (dishevelled; dsh homolog 2 (Drosophila))  0.00228326 CNIH4 (cornichon homolog 4 (Drosophila)) |
| *COL23A1* | rs17081346 | NA | 1.1551E-05 TMEM97 (transmembrane protein 97)  8.3426E-05 TAX1BP1 (Tax1 (human T-cell leukemia virus type I) binding protein 1)  0.00013049 HS.562660  0.00017336 MTHFS (5;10-methenyltetrahydrofolate synthetase (5-formyltetrahydrofolate cyclo-ligase))  0.00028995 ABTB1 (ankyrin repeat and BTB (POZ) domain containing 1)  0.00032666 CHD2 (chromodomain helicase DNA binding protein 2)  0.00034791 RNASEH1 (ribonuclease H1)  0.00052212 MAGMAS (mitochondria-associated protein involved in granulocyte-macrophage colony-stimulating factor signal transduction)  0.00055235 CD36 (CD36 molecule (thrombospondin receptor))  0.00060783 PINK1 (PTEN induced putative kinase 1) |
| *RAMP1* | rs3769047 | NA | 5.2994E-06 CLDN9 (claudin 9)  7.6395E-06 OR4B1 (olfactory receptor; family 4; subfamily B; member 1)  2.5671E-05 MTRF1L (mitochondrial translational release factor 1-lik)  2.7402E-05 HS.574766  4.0538E-05 HS.339163  0.00010504 CFH (complement factor H)  0.00013536 MAPRE2 (microtubule-associated protein; RP/EB family; member 2)  0.00013645 BTBD14A (BTB (POZ) domain containing 14A)  0.00017537 FGF23 (fibroblast growth factor 23)  0.00018985 FLJ13224 |

*adjusted for sex, age, race, extraction batch
